# Self-Relevance Predicts the Aesthetic Appeal of Real and Synthetic Artworks Generated via Neural Style Transfer

**DOI:** 10.1177/09567976231188107

**Published:** 2023-08-14

**Authors:** Edward A. Vessel, Laura Pasqualette, Cem Uran, Sarah Koldehoff, Giacomo Bignardi, Martin Vinck

**Affiliations:** 1 Department of Neuroscience, Max Planck Institute for Empirical Aesthetics; 2 Neurocognitive Developmental Psychology, Friedrich-Alexander University Erlangen-Nürnberg; 3 Ernst Strüngmann Institute; 4 Department of Neurophysics, Donders Centre for Neuroscience; 5 Department of Language and Genetics, Max Planck Institute for Psycholinguistics; 6 Max Planck School of Cognition

**Keywords:** aesthetic valuation, artwork, identity, machine learning, open data

## Abstract

What determines the aesthetic appeal of artworks? Recent work suggests that aesthetic appeal can, to some extent, be predicted from a visual artwork’s image features. Yet a large fraction of variance in aesthetic ratings remains unexplained and may relate to individual preferences. We hypothesized that an artwork’s aesthetic appeal depends strongly on self-relevance. In a first study (*N* = 33 adults, online replication *N* = 208), rated aesthetic appeal for real artworks was positively predicted by rated self-relevance. In a second experiment (*N* = 45 online), we created synthetic, self-relevant artworks using deep neural networks that transferred the style of existing artworks to photographs. Style transfer was applied to self-relevant photographs selected to reflect participant-specific attributes such as autobiographical memories. Self-relevant, synthetic artworks were rated as more aesthetically appealing than matched control images, at a level similar to human-made artworks. Thus, self-relevance is a key determinant of aesthetic appeal, independent of artistic skill and image features.

Experiences with artwork can impact us deeply. They can move us emotionally (Menninghaus et al., 2019) and inspire us creatively (Welke et al., 2023). They can also be transformative, opening up new ways of looking at ourselves and the world around us. What determines an artwork’s aesthetic appeal and differences in aesthetic experiences between individuals?

Whereas aesthetic judgments of faces and natural landscapes tend to be relatively consistent across individuals, studies suggest that “shared taste” accounts for only 10% to 20% of reliable variance in aesthetic ratings of artworks (Leder et al., 2016; Vessel et al., 2018). Importantly, shared taste represents a ceiling for how much variance in aesthetic appeal can be accounted for by any set of intrinsic stimulus properties. A wide variety of image properties have been tested for their ability to predict aesthetic ratings of artwork (for a review, see Vessel et al., 2022). A recent report indicated that a linear combination of low- and high-level image features could predict approximately 20% of variance in aesthetic ratings of artwork (Iigaya et al., 2021). A comparable performance (16.4%) was obtained by a deep neural network trained to predict aesthetic ratings (Iigaya et al., 2021). The performance of these predictive models is at the theoretical limit imposed by the shared taste ceiling for artwork (Conwell et al., 2021). The remaining 80% to 90% of variance in aesthetic ratings differs from person to person and remains unexplained.

We hypothesized that a key factor explaining aesthetic appeal of art is an artwork’s capacity to resonate with a person depending on whether it speaks to one’s individually lived experience. Does the artwork resonate with one’s sense of self and worldview? This can be captured by the concept of self-relevance, which reflects the extent to which something relates to a person’s self-schema: their self-perception, past experiences, and personal and social identity (Wagner et al., 2012). Social psychology studies have shown that one’s self-schema is chronically accessible (Markus, 1977), is accessed when other individuals are evaluated (Markus et al., 1985), and affects memory encoding (Klein & Loftus, 1988; Rogers et al., 1977). Because artworks are communicative objects that reflect other people’s thoughts and intentions (Goodman, 1976; Menninghaus et al., 2017), their evaluation may also involve access of the self-schema. Indeed, several frameworks for understanding aesthetic experiences suggest that self-relevance is central to aesthetic evaluations of artwork (Pelowski et al., 2017). Furthermore, brain-imaging studies suggest that the default-mode network, which supports central aspects of self-referential mentation (Andrews-Hanna et al., 2010; D’Argembeau et al., 2010; Moran et al., 2009), plays a role in aesthetically moving experiences with artwork (Vessel et al., 2013, 2019). Yet it is not obvious that self-relevance should necessarily be a predictor of aesthetic appeal; artworks that serve as vehicles for understanding the experiences of other people can also be highly moving. In addition, aspects of one’s self-construct are not necessarily positively valenced, and emotional intensity predicts the properties of autobiographical memories more than positive valence (Talarico et al., 2004).

We thus performed two sets of experiments to directly investigate the influence of self-relevance on aesthetic appeal. In a first observational study and replication, we observed a strong correlation between self-relevance assessments and aesthetic ratings. In a second experiment, we developed a new method to manipulate self-relevance by using a deep-learning technique known as *style transfer* (Gatys et al., 2016). By transferring the styles of existing artworks to pictures using neural networks, we created novel artworks that related to each participant’s unique self-schema: their autobiographical memories, identity, and interests. These were then rated by participants for aesthetic appeal, along with artworks created for a different observer and a set of real artworks. We then analyzed which aspects of self-relevance accounted for the observed effects on aesthetic appeal.

**Statement of Relevance**

Experiences with art can be transformative. Yet even for our everyday experience, aesthetic factors strongly influence behavior, mood, and productivity, and the ability to predict people’s tastes is central to the business model of many successful companies. We used a machine-learning algorithm called *style transfer* to generate novel artworks with custom-tailored content that reflected individuals’ self-construct: their memories, their interests, and their identity. Our results reveal a tight connection between aesthetic appeal and one’s sense of self: For visual artwork, a connection to one’s lived experience is actually more predictive of its impact than any directly measurable feature of the art itself. Given the increasing presence of algorithms that attempt to predict what we like and deliver personalized content on the basis of personal information, it is critical to study the psychological impact of such content and understand its potential for both use and abuse.

## Method

### Experiment 1A

#### Participants

There exists no previous estimate of the relationship between self-relevance and aesthetic appeal. To derive an initial estimate of this relationship, we conducted an initial study using a convenience sample of 33 German-speaking participants (29 female, four male; 30 right handed, three left handed) between the ages of 18 and 55 years (age: *M* = 28.9 years, *SD* = 7.3) who were recruited through a research participant database maintained by the Max Planck Institute for Empirical Aesthetics and by advertisements on the institute website (see the Data Analysis section for post hoc power analysis). Participants had normal or corrected-to-normal vision and no known neurological disorders. They were informed that the study would be about rating artworks according to how pleasing those pieces were for them; they were not previously aware of the study’s interest in self-relevance. Participants signed a consent form and received monetary compensation. All recruitment and study procedures were approved by the Ethics Council of the Max Planck Society.

#### Stimuli

Images and instructions were presented on an ASUS VG248QE Full-HD Gaming Monitor (60.96 cm) with a computer running Windows 10 Pro (Microsoft Corporation, Redmond, WA) and MATLAB R2017a (The MathWorks, Natick, MA) with Psychophysics Toolbox–3 extensions (Brainard, 1997; http://psychtoolbox.org). Participants were positioned approximately 57 cm from the monitor.

The stimulus set consisted of 148 photos of visual artworks used in a previous study (Vessel et al., 2018). The images were originally selected from the Catalog of Art Images Online (note that this database was closed in December 2018 and is no longer accessible). The images were taken from museum collections, although commonly reproduced artworks were not included to minimize the possibility of recognition. Moreover, the set covered a variety of time periods, styles, and genres from cultures across the Americas, Europe, and Asia (see Vessel et al., 2018, 2019). For the full list of artworks, see the Supplemental Material available online. Images were scaled such that the largest dimension did not exceed 20° of visual angle, and the area did not exceed 75% of a 20° box.

For the retest sessions, 20 representative images were selected from the larger set of 148. The practice test used 10 additional images not contained in the primary stimulus set.

#### Procedure

Following the informed consent procedure, participants were brought into a computer booth and instructed to sit comfortably in front of the screen and mouse. The whole experiment consisted of four blocks. Before each block, observers were given written and verbal instructions for that block. Block 1 consisted of aesthetic ratings of all 148 artworks (in random order, but with even-numbered observers getting the reverse order of odd-numbered participants). Block 2 consisted of self-relevance ratings of all 148 artworks (same order as for Block 1). Observers were given 10 practice trials before Blocks 1 and 2 to familiarize themselves with the task and general nature of the artworks. Blocks 3 and 4 were retest blocks for aesthetic ratings and self-relevance ratings, for a subset of 20 artworks. Within as well as between blocks, participants were allowed breaks.

Each trial began with a fixation cross for 0.5 s, followed by the artwork for 5 s and then the response screen, which remained until the participant locked in his or her response by clicking the mouse button. Response time was unconstrained. A mid-gray background was used for all phases of the experiment.

#### Ratings for aesthetic appeal

Participants were asked to rate how aesthetically pleasing an image was for them using a continuous response scale. Participants were encouraged to answer according to their own aesthetic evaluation on the basis of how “moved” they were by the paintings. Observers used the mouse to move a slider that appeared on the screen, marked at the ends with “N” (for “Niedrig,” meaning low) and “H” (for “Hoch,” meaning high), and were encouraged to use the full range of the scale. For the full instructions, see the Supplemental Material.

#### Ratings for self-relevance

Participants were asked to rate how self-relevant an artwork was for them using a continuous response scale. Self-relevance was defined as “the extent to which something relates to you, your experiences, or your identity. These are the things and events that define you as a person.” Observers used the mouse to move a slider that appeared on the screen, marked at the ends with “N” (for “Niedrig,” meaning low) and “H” (for “Hoch,” meaning high), and were encouraged to use the full range of the scale. For the full instructions, see the Supplemental Material.

#### Questionnaires

Before the experiment, participants completed a questionnaire assessing basic demographic information (education background, age, gender, handedness, sexual orientation, diagnosed mood/psychological/neurological disorders, medication) and further education in the field of arts and aesthetics. In addition, participants completed the Aesthetic Responsiveness Assessment (Schlotz et al., 2021), a short questionnaire that identifies three major aspects of an individual’s responsiveness to aesthetic experiences; the Big Five Inventory–Extra Short Form (Soto & John, 2017), which measures the Big Five personality traits; and the abbreviated Positive and Negative Affect Schedule (Watson & Clark, 1999), which was used to measure state affect. The Positive and Negative Affect Schedule was applied also after experiment conduction from Participant 5 onward.

#### Data analysis

Data were analyzed and visualized in the R programming environment (Version 4.2.2; R Core Team, 2022). Slider responses for both aesthetic appeal and self-relevance were coded on a scale from 0 to 1.

A reliability score was computed for both aesthetic and self-relevance ratings as the correlation between ratings of the 20 artworks shown in both test and retest blocks, separately for each participant. These two scores were then transformed to *z* scores (Fisher *r* to *z*), averaged, and transformed back to *r* values to produce an average reliability score for each participant. One observer’s average reliability score was below the 0.5 cutoff value and was thus removed from further analysis (final *N* = 32).

A linear mixed model (LMM) using *lmer* from the *lme4* package in R (Bates et al., 2015) was computed to predict ratings of aesthetic appeal (Block 1) from ratings of self-relevance (Block 2). Models were computed that included only participant-specific intercepts (Model 1), AesthRatings~SelfRelevRatings​(1|Participant) ; participant-specific intercepts plus slopes (Model 2), AesthRatings~SelfRelevRatings​(SelfRelevRatings|Participant) ; participant-specific intercepts and slopes plus image-specific intercepts (Model 3), AesthRatings~SR​(SelfRelevRatings|Participant)​(1|Image) ; and participant-specific intercepts and slopes plus image-specific intercepts and slopes (Model 4), AesthRatings~SelfRelevRatings​(SelfRelevRatings|Participant)​(SelfRelevRatings|Image) . Model comparison using Akaike information criterion (AIC) measures (*anova* function) revealed that whereas Model 3 clearly outperformed the others—log likelihood M1 = 506, M2 = 609, M3 = 787, M3 versus M2, $\chi^{2}(1)=358$ , $p<2\times 10^{-16}$ —the addition of image-specific slopes did not lead to a significant improvement in fit—log likelihood M4 = 791, M4 versus M3, $\chi^{2}(2)=5.1$ , *p* = .08. Degrees of freedom for *t* tests were computed using Satterthwaite’s method (*lmerTest*; Kuznetsova et al., 2017), and 95% confidence intervals (CIs) were computed by bootstrapping with 500 simulations using the *confint.merMod* function from *lme4*. Effect sizes (partial $\eta^{2}$ ) were computed using the *effectsize* function.

A post hoc power analysis using G*Power (Version 3.1; Faul et al., 2009) indicated that a sample of 32 participants rating 148 images (total of 4,736 measurements), modeled using random predictors for participant intercepts, participant slopes, and item intercepts (213 total predictors), resulted in a power (β) of 0.89 to detect an overall $R^{2}$ of .0625 (e.g., correlation of r=.25 , corresponding to $\rho^{2}=.0193$ , calculated using a 95% CI) at an alpha of .01. The experiment was thus well powered to detect even smaller associations between self-relevance and aesthetic appeal.

To quantify the degree of shared taste and shared self-relevance across observers, we computed leave-one-out (or “mean-minus-one”) agreement scores following the procedure by Vessel et al. (2018). For each participant, a Pearson correlation was computed between their set of ratings for the image set and the average of all other observers. These individual correlations were then converted to *z* scores (*r*-to-*z* transform), averaged, and converted back into *r* scores.

Variance partitioning was conducted by first computing a multilevel model (using *lmer*) with random intercepts for stimuli, participants, block (test vs. retest), and the two-way interactions between these terms (Martinez et al., 2020). The resulting variance partitioning coefficients (VPCs) were then combined to compute the proportion of repeatable variance that is individual versus shared (Martinez et al., 2020), where

$$\sigma_{repeat}^{2}=\sigma_{tot}^{2}-\sigma_{error}^{2}$$

is the repeatable variance,

$$\begin{array}{l} VPC_{particip}=\frac{\sigma_{particip}^{2}}{\sigma_{repeat}^{2}};VPC_{stimXparticip}=\frac{\sigma_{stimXparticip}^{2}}{\sigma_{repeat}^{2}};\\ \;\;\;VPC_{participXblock}=\frac{\sigma_{participXblock}^{2}}{\sigma_{repeat}^{2}} \end{array}$$

are the VPCs,

$$\sigma_{indiv}^{2}=VPC_{particip}VPC_{stimXparticip}VPC_{participXblock}$$

is the variance that is “individual,” and

$$\sigma_{shared}^{2}=1-\sigma_{indiv}^{2}$$

is the variance that is “shared.”

For details of the intrinsic image property analysis, see the Supplemental Material.

### Experiment 1B

#### Participants

Because of COVID-19 restrictions and the need to collect a large participant pool, this experiment was conducted online. We aimed for a total sample of more than 200 participants (see below for sensitivity analysis), and 243 English-speaking participants were recruited online via Prolific (Oxford, UK). As elaborated below, data from 35 participants were excluded from analysis, resulting in a final sample of 208 participants (135 male, 70 female, two identified as other gender, one did not state gender; age: range = 18–74 years, *M* = 27.3 years, *SD* = 9.3). Using Prolific filters, we selected participants who were fluent in English, had normal or corrected-to-normal vision, and had no untreated mental or psychiatric illness. They were informed that the study would be about rating artworks according to how pleasing those pieces were for them; they were not previously aware of the study’s interest in self-relevance. Participants digitally signed a consent form and received monetary compensation. All recruitment and study procedures were approved by the Ethics Council of the Max Planck Society.

#### Stimuli

Each participant viewed 42 artworks, which were a subset of those used in Experiment 1A. This subset was selected to represent the diversity of styles, genres, periods, and content present in the full set as best as possible, with advice from a consulting art historian. For the full list of artworks, see the Supplemental Material. All images were scaled such that the longer dimension was 600 pixels.

#### Procedure

From Prolific, participants were directed to a JATOS server (https://www.jatos.org) hosted at the Max Planck Institute for Empirical Aesthetics. Demographic information and a set of personality measures for a separate experiment were collected using a script created from SurveyJS templates (https://surveyjs.io). These surveys included one item that required participants to select a specific response as an attention check.

The main experiment, coded using the OpenSesame experiment builder (Mathôt et al., 2012; https://osdoc.cogsci.nl), consisted of a set of general instructions and two blocks of trials. The experimental window was set to a resolution of 1,280 pixels × 720 pixels, and participants were instructed to view this window in full-screen mode. Each block began with a set of specific instructions and three practice trials. In the first block, participants viewed each image for 5 s, followed by a screen on which they were required to rate each image on 10 different questions. Participants responded by using the mouse to click on a location on a continuous slider bar for each question. A smaller version of the image was presented next to the rating scales. When all 10 questions had been answered, the participant clicked a “next” button to proceed to the next trial. For this experiment, we focused on two of these questions, which we refer to as “being moved” (“To what extent did the image move you?”) and “beauty” (“How much did you get the feeling of beauty?”). Analyses of additional questions and personality measures are the focus of a separate, forthcoming report.

In the second block, participants saw each artwork again in a new random order and responded to the question, “How self-relevant is the image to you?” This single question and a continuous response scale were presented below the artwork, which remained on the screen until the participant used the mouse to answer and clicked “next” to proceed. For both blocks, two artworks were repeated to assess test-retest reliability (retest artworks are indicated in the List of Artworks in the Supplemental Material).

#### Data analysis

Data were analyzed and visualized using R (R Core Team, 2022). Slider responses were initially collected on a 400-point interval scale and subsequently recoded to values between 0 and 1.

A reliability score was determined for each participant by computing a correlation between the set of 11 ratings given for the first and second presentations of each of the two repeated images and then averaging these two values. Following examination of the distribution of these scores, we adopted a conservative cutoff of an average of r<.4 . This resulted in the exclusion of 31 participants. Nine participants failed the attention check, four of whom were in addition to those already excluded (final N=208 ).

LMMs were computed to predict beauty and being-moved ratings from self-relevance, using the same procedure and models as in Experiment 1A. Model comparison using AIC revealed that for beauty, Model 4, AesthRatings~SelfRelevRatings​(SelfRelevRatings|Participant)​(SelfRelevRatings|Image) , outperformed the others—log likelihood M1 = −283, M2 = −142, M3 = 1055, M4 = 1068, M4 versus M3, $\chi^{2}(2)=25.0$ , $p<4\times 10^{-6}$ . For being moved, Model 4 also performed best—log likelihood M1 = 627, M2 = 832, M3 = 1592, M4 = 1603, M4 versus M3, $\chi^{2}(2)=22.5$ , $p<1.5\times 10^{-5}$ .

A sensitivity analysis using G*Power (Version 3.1; Faul et al., 2009) indicated that a sample of 208 participants rating 42 images (total of 8,736 measurements), modeled using random predictors for participant intercepts, participant slopes, and item intercepts (459 total predictors), was sensitive to detect an overall $R^{2}$ of .065 (e.g., correlation of r=.256 , corresponding to $\rho^{2}=.0140$ , calculated using a 95% CI) at a power (β) of 0.90 and alpha of .01. The experiment was thus well powered to detect very small associations between self-relevance and aesthetic appeal.

### Experiment 2

#### Participants

On the basis of the partial $\eta^{2}$ of .83 computed from Experiment 1A, we might expect a large effect of self-relevance on aesthetic appeal. We conducted a power analysis using G*Power (Version 3.1; Faul et al., 2009) for a two-tailed paired-samples *t* test (power β = 0.8, α = .05) of the self-relevant versus other-relevant conditions. To detect a small effect size (*d*) of 0.1, we would need 787 samples. With 20 items per condition, we thus aimed to collect a final sample of 40 participants, resulting in 800 trials per condition. We recruited 59 participants online via Prolific for the initial session (see below), of which 52 were invited to take part in the main session, and 45 completed. Participants not selected for the second session were excluded because they did not provide enough answers to the Cultural Background and Lifestyle Questionnaire (see below) to allow for self-relevant stimulus creation. The participants were between the ages of 18 and 55 years (28 male, 15 female, two nonidentified; 41 right handed, three left handed, one ambidextrous; age: *M* = 29.8 years, *SD* = 8.0), were fluent in German, had normal or corrected-to-normal vision, and had no previously known neurological disorders. They were informed that the study would be about rating artworks according to how pleasing those pieces were for them; they were not previously aware of the study’s interest in self-relevance. Participants digitally signed a consent form and received monetary compensation. All recruitment and study procedures were approved by the Ethics Council of the Max Planck Society.

#### Stimuli

Each participant viewed 80 artworks, 20 in each of four conditions. The real artworks condition consisted of 20 paintings selected from the set used in Experiment 1A to cover a variety of time periods, styles, genres, and cultural origin. For the full list of artworks, see the Supplemental Material. The remaining three conditions consisted of novel artworks generated using a style-transfer algorithm (see below).

The generated-control artworks were the same for all participants. Generated-control content source photographs contained a mixture of natural and manufactured content, outdoor and indoor scenes, and built structures and objects.

The self-relevant artworks were generated uniquely for each participant. Content source images were collected from online sources (Google Images, open source websites; minimum size of 800 pixels × 600 pixels) that depicted places, objects, cuisine, animals, and cultural artifacts referenced by the participant in the Cultural Background and Lifestyle Questionnaire.

The other-relevant artworks consisted of the set of self-relevant artworks generated for a paired participant. The applied artistic styles (but not source content) were matched across the two paired participants to control for the effect of style.

An initial set of 96 artworks was identified as potential style source images. In addition to the set of artworks used in Experiment 1A, additional artworks were gathered from online collections of the Metropolitan Museum of Art (metmuseum.org), the National Gallery of Art (nga.gov), and the Rijksmuseum (rijksmuseum.nl). Of these, 56 were used as style sources for the generated-control, self-relevant, and other-relevant conditions.

The images used for practice trials were a mix of real artworks and style-transferred artworks, different from those used in the main experiment.

#### Style transfer

Novel generated artworks were created using the adaptive instance normalization method developed by Huang and Belongie (2017). Like the original style-transfer method (Gatys et al., 2016), it uses a deep convolutional neural network to apply the style of an artwork to a photograph. The general approach is to match the low- and mid-level feature statistics of the input style across the hierarchical feature representations (convolutional layers) of the deep convolutional neural network-encoded content image. The network accomplishes this by first encoding both the content and style images in the feature space of an encoder network composed of the first few layers of a pretrained VGG-19 (Visual Geometry Group, Oxford, UK) deep convolutional neural network. The feature maps are then fed to the adaptive instance normalization layer, which aligns the mean and variance of the content feature maps to those of the style feature maps to produce a set of target feature maps. Finally, a decoder (randomly initialized) is trained to map from the target feature map back into image space, which generates a stylized image.

For each pair of observers, the two sets of 20 self-relevant content images were combined with several input styles to identify viable combinations that could be applied to an image from each of the two sets without introducing excessive blur or artifacts. Our goal was to generate images that looked, as much as possible, like actual artworks. Some styles were found to work better for certain types of content (landscape, object closeup, building, etc.) and were used more frequently. However, styles were always matched across the pair of observers, with any one style being used for only one pair of images in each pair of observers. The size of the output images was matched to the input size of the photograph, and all images were saved in jpeg format.

#### Procedure

On account of COVID-19 related restrictions, data were collected using the web-based Unipark Survey Platform (QuestBack GmbH, Cologne, Germany). The experiment consisted of two sessions and was conducted in German. In Session 1, participants completed a set of questionnaires that included the Cultural Background and Lifestyle Questionnaire used to identify self-relevant content (see below). Participants were then grouped into pairs for the generation of self-relevant and other-relevant images, making sure that their responses on this questionnaire were sufficiently divergent.

Participants invited to Session 2 answered several questionnaires and then completed four experimental blocks. In Block 1, participants rated the full set of 80 artworks for aesthetic appeal (identical instructions as in Experiment 1A). In Block 2, participants rated the full set of artworks for aesthetic appeal a second time to assess test-retest reliability. In Block 3, participants rated the full set of artworks for self-relevance (identical instructions as in Experiment 1A). In Block 4, participants rated the full set of artworks for familiarity. Stimuli were presented in a pseudorandom order that distributed the four conditions evenly across the entire block and balanced the one-back trial history of each condition to minimize any serial order effects. No more than two repetitions of each condition were allowed in a sequence. The same order was used for all four blocks. Even-numbered participants were shown condition orders that were a reversal of orders for odd-numbered participants to control for the average serial position of each condition.

Each trial began with a fixation cross for 1 s, followed by the artwork for 4 s and then the response screen. A mid-gray background was used for all phases of the experiment.

#### Cultural Background and Lifestyle Questionnaire

To identify candidate content for the creation of self-relevant images, we designed a 20-item Cultural Background and Lifestyle Questionnaire that asked participants about a variety of significant locations associated with autobiographical experiences, aspects of their personal identity, and personal interests in topics such as the arts, style, and cuisine (for the full questionnaire, see the Supplemental Material).

#### Additional questionnaires

During the first session, participants completed a demographic background questionnaire, the Aesthetic Responsiveness Assessment (Schlotz et al., 2021), and the Big Five Inventory 2–Short Form (Soto & John, 2017). During the second session, participants completed the abbreviated Positive and Negative Affect Schedule (Watson & Clark, 1999) and both the trait and state forms of the State-Trait Anxiety Inventory (Spielberger et al., 1983).

#### Familiarity judgment

Participants were asked to indicate whether each artwork was unfamiliar, familiar, or definitely recognized by clicking a checkbox. Because the images had already been seen several times during the experiment, the instructions emphasized that they were to answer on the basis of their knowledge prior to the experiment. Observers were to choose “definitely recognize” if they knew for certain that they had seen the artwork (or an image of it) before, with high confidence. They were to choose “familiar” if they had a sense that they had seen the artwork (or an image of it) before or that the content felt familiar. They were to choose “unfamiliar” if they had high confidence that they had not seen the artwork before.

We note that participants were not told beforehand that the artworks they were viewing were artificially generated. For these paintings, it was not actually possible for the image to have been seen previously, and hence they were all novel in a strict sense (unlike for the real paintings, where it was possible, although unlikely, that they had been previously seen). Thus, the wording of our instructions was constructed in a manner that allowed observers to express a feeling of familiarity with the content. Instances in which the observer found the content familiar or even explicitly recognizable (e.g., a place they had been before) could thus be reasonably marked “familiar” without the observer having to reconstruct the source of that familiarity. For the full instructions, see the Supplemental Material.

#### Data analysis

Data were analyzed and visualized using R (R Core Team, 2022) and MATLAB (The MathWorks, Natick, MA). Slider responses for both aesthetic appeal and self-relevance were coded on a scale from 0 to 100 and subsequently rescaled to between 0 and 1.

A test-retest score was computed (Pearson correlation) for each participant on the basis of the first and second blocks of aesthetic ratings. Five participants with test-retest scores of r<.5 were excluded from further analysis (final N=40 ; for additional information, see the Supplemental Material).

LMMs for categorical effects were computed using *lmer* from the *lme4* package in R (Bates et al., 2015) with three planned contrasts (self-relevant vs. other relevant, real artworks vs. generated-control, and self-relevant vs. real artworks) and degrees of freedom for *t* tests computed using Satterthwaite’s method, AesthRatings~Condition​(1|Participant) ; SelfRelevRatings~Condition​(1|Participant) . Additional post hoc comparisons (e.g., other relevant vs. generated-control) were computed as Tukey contrasts using *glht* from the *multcomp* package, with adjusted *p* values computed using the Holm method. CIs were computed by bootstrapping with 500 simulations using the *confint.merMod* function from *lme4*. Cohen’s *d* scores were computed by dividing the estimate of each contrast by the pooled standard deviation of the random effects. For the purpose of plotting the condition averages in Figure 3, a second set of models was computed using participant-centered data.

The LMM for prediction of aesthetic appeal from ratings of self-relevance and familiarity was conducted using *lmer* on ratings rescaled to between 0 and 1. Two participants were excluded because they completed the familiarity task incorrectly, rating the images on the basis of whether they had been seen during the session, rather than before the session (task instructions were subsequently modified to prevent this misunderstanding). First, a model was run predicting aesthetic appeal from self-relevance alone, with random intercepts for participants and condition (Model 1), AesthRatings~SelfRelevRatings​(1|Condition) . We then computed two additional models using *lmer* predicting aesthetic appeal from ratings of self-relevance and familiarity, with random intercepts for participant and condition, both without random slopes (Model 2), AesthRatings~SelfRelevRatings​Familiarity​(1|Participant)​(1|Condition) , and with random slopes (Model 3), AesthRatings~SelfRelevRatings​Familiarity​(SelfRelevRatings|Participant)​(SelfRelevRatings|Condition) , for self-relevance by condition and participant. Familiarity was coded using two orthogonal contrasts: “definitely recognize” and “familiar” versus “unfamiliar” (RF vs. U) and “definitely recognize” versus “familiar” (R vs. F). We note that any image rated as recognized is by definition also familiar. Model comparison using AIC measures from *anova* revealed that the full model including both self-relevance and familiarity and random slopes for self-relevance by condition and participant performed best—Model 2 vs. Model 1 log likelihood 272 vs. 243, $\chi^{2}(2)=57$ , $p=2.7\times 10^{-13}$ ; Model 3 vs. Model 2 log likelihood 332 vs. 272, $\chi^{2}(4)=119$ , $p<2.2\times 10^{-16}$ .

A formal mediation analysis was then computed using the *mediation* package in R to test whether the effect of self-relevance on aesthetic appeal was mediated by familiarity, with CIs computed using nonparametric bootstrapping with 1,000 simulations.

#### Subclasses of self-relevance

The 20 questions from the Cultural Background and Lifestyle Questionnaire were assigned to one of five subgroups on the basis of the nature of information sought by the question: specific autobiographical memories (238 items), aspects of personal identity (26 items), interests (86 items), common activities (39 items), and explicitly expressed preferences (e.g., “My favorite food is Vietnamese pho soup”; 87 items). Because of their wording, some questions could not be unambiguously assigned to one of these categories and were thus assigned to a sixth “mixed” class (324 items). An LMM was conducted using *lmer* to predict aesthetic appeal for the 20 generated artworks in the self-relevant condition as a function of question class, along with participant-specific intercepts; ratings for the 20 control-generated images were included as a baseline comparison and are thus reflected in the model intercept (800 items total).

### Learned Perceptual Image Patch Similarity (LPIPS)

LPIPS is a perceptual similarity metric based on convolutional neural network activations (see Uran et al., 2022). It was computed on the basis of the VGG-16 model without the learned weights, which is the average content loss (L2 distance) of layers conv1_2, conv2_2, conv3_3, conv4_3, and conv5_3. Pearson correlation *p* values were computed using a Student’s *t* distribution for a transformation of the correlation.

## Results

### Ratings of self-relevance strongly predict aesthetic appeal

In Experiment 1A, 33 participants viewed 148 artworks and rated them for aesthetic appeal (“How much does this artwork move you?”) and self-relevance (“How self-relevant is this artwork for you?”) in separate blocks. Twenty of the artworks were viewed and rated again on both measures to allow for computation of participant-specific test-retest reliability scores and estimation of repeatable variance (75% and 83% repeatable variance for aesthetic appeal and self-relevance, respectively).

**Fig. 1.**
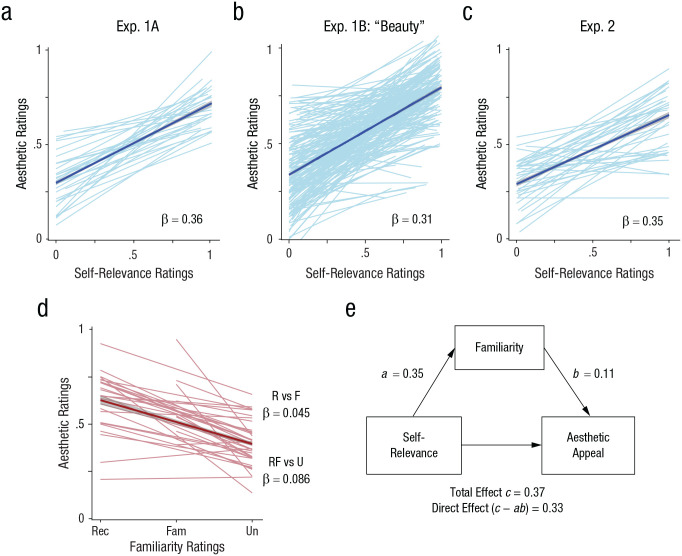
Predicting aesthetic ratings from ratings of self-relevance and familiarity. (a) For Experiment (Exp.) 1A, self-relevance ratings predicted aesthetic appeal with an average slope of 0.36 (*N* = 32). (b) For Experiment 1B, self-relevance ratings predicted aesthetic appeal (“feeling of beauty”) with an average slope of 0.31 (*N* = 208). (c) For Experiment 2, self-relevance ratings predicted aesthetic appeal with an average slope of 0.35 (*N* = 40). (d) In Experiment 2, familiarity also predicted ratings of aesthetic appeal, with the larger effect for ratings of recognized (R) or familiar (F) artworks versus unfamiliar (U) artworks. There was a smaller but significant effect for recognized versus familiar artworks. Rec = definitely recognized; Fam = familiar; Un = unknown (*N* = 40). (e) A mediation model of Experiment 2 data shows that the total effect of self-relevance on aesthetic appeal *c* was only partially mediated by familiarity (paths *a* and *b*), leaving a strong direct effect (*c – ab*). Thick dark blue (a–c) and red (d) lines indicate the average linear slope (with the standard error of the estimated slope in gray), and the thin light blue (a–c) and pink (d) lines show linear slopes for individual participants.

Individual ratings of aesthetic appeal were modeled as a function of individual ratings of self-relevance using an LMM, with participant-specific intercepts and slopes and image-specific intercepts (see the Method section). Individual ratings of whether a painting was judged to be self-relevant were strongly predictive of aesthetic ratings (Fig. 1a), slope = 0.36, 95% CI = [0.30, 0.42], *t*(32.3) = 12.4, *p* = 8.3 × 10^-14^, partial $\eta^{2}=.83$ , despite the fact that observers differed in which paintings they found to be aesthetically appealing or self-relevant (mean-minus-one agreement metric MM1=0.40 and 0.39 , respectively; 19% and 18% of repeatable variance explained by shared taste or shared self-relevance). This relationship was even stronger in the retest block, slope = 0.62, 95% CI = [0.53, 0.70], *t*(30.2) = 14.9, *p* = 1.9 × 10^-15^, partial $\eta^{2}=.88$ , suggesting that calling explicit attention to the dimension of self-relevance further increased the already strong relationship between these two constructs (although we note that retest blocks included only a subset of 20 images).

Despite the low agreement across individuals for ratings of both aesthetic appeal and self-relevance, it remained possible that a portion of this variance was related to the presence of specific image features. A set of 15 image features derived from models of visual aesthetics (Redies et al., 2020) and natural image encoding (Uran et al., 2022), along with scores for image memorability (Needell & Bainbridge, 2022), was computed for each artwork (see the Supplemental Material). We also collected ratings of naturalness and disorder, key factors for scene preferences (Kotabe et al., 2017), from an independent set of raters (see the Supplemental Material). Crucially, the strength of the relationship between self-relevance and aesthetic appeal varied only trivially when accounting for these intrinsic image properties (see Fig. S1b and Table S1 in the Supplemental Material), slope = 0.35, 95% CI = [0.30, 0.42], *t*(32.3) = 12.3, *p* = 9.22 × 10^-14^, partial $\eta^{2}=.82$ . Using a variance partitioning approach (see Fig. S1c in the Supplemental Material), we found that these image factors, considered on their own, explained approximately 8% of the variance in aesthetic ratings, or slightly more than half of the stimulus-related (shared) variance. Self-relevance, when considered on its own, accounted for approximately 7% variance that was stimulus related, approximately 5% that was participant related, and approximately 16% that was related to the interaction of stimulus and participant. Self-relevance was thus able to predict approximately 28% of the total variance in aesthetic appeal, of which only a fraction (≈5%) overlapped with the image feature model (see Fig. S1d in the Supplemental Material). Even when one looks only at average aesthetic ratings (reflecting shared taste), self-relevance was dominant over any of the features of the image model (see the Supplemental Material).

Given the small sample size of this in-person study, we then replicated the effect in Experiment 1B, an online study in which 208 participants viewed and rated 42 artworks for the feeling of beauty, being moved, and several other factors in a first block, and self-relevance in a second block. Individual ratings of self-relevance were again strongly predictive of aesthetic ratings of both beauty, slope = 0.31, 95% CI = [0.28, 0.34], *t*(176.3) = 19.9, *p* = 4.7 × 10^-47^, partial $\eta^{2}=.69$ (Fig. 1b), and being moved, slope = 0.25, 95% CI = [0.22, 0.28], *t*(154.5) = 15.2, *p* = 1.4 × 10^-32^, partial $\eta^{2}=.60$ (see Fig. S2 in the Supplemental Material). For both Experiments 1A and 1B, local (loess) fits suggested that whereas there may be a degree of nonlinearity in their relationship, aesthetic appeal increased monotonically across the entire range of self-relevance (see Fig. S3 in the Supplemental Material).

### Self-relevant artworks generated using neural style transfer are more aesthetically appealing than matched artworks generated for other individuals

**Fig. 2.**
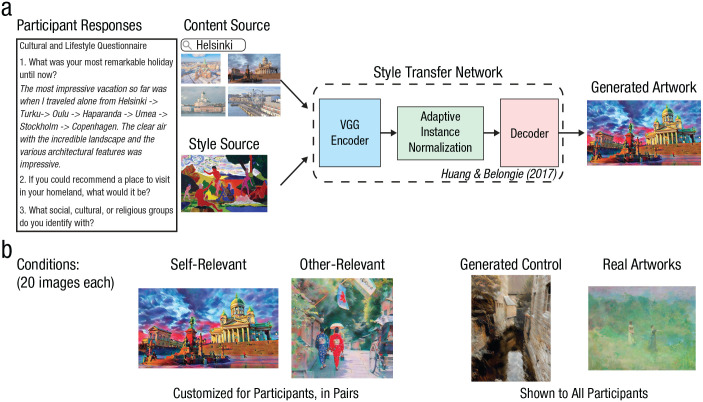
Generating self-relevant artworks using a “style transfer” convolutional neural network. (a) In a first session, participants responded to a Cultural Background and Lifestyle Questionnaire that inquired about specific autobiographical memories, aspects of identity, interests, preferences, and common activities. Images that contained content relevant to each observer’s individual responses were then sourced from the Internet. A style of an existing artwork was then transferred to the image using a style-transfer network based on the work by Huang and Belongie (2017) that consisted of an encoder network, adaptive instance normalization, and a decoder network, resulting in a new synthetic artwork with customized content. (b) In a second session, observers were shown artworks from four conditions: (1) self-relevant artworks custom generated on the basis of their questionnaire responses, (2) other-relevant artworks generated for a matched participant, (3) a control set of generated artworks, and (4) a set of real artworks. There were 20 artworks in each condition. VGG = Visual Geometry Group, Oxford, UK. Real Artwork credit: Bob Thompson (1937–1966), *Homage to Nina Simone*, 1965, oil on canvas, 48 × 72 1/8 inches; Collection of The Minneapolis Institute of Art; © Michael Rosenfeld Gallery LLC, New York, NY; Courtesy of Michael Rosenfeld Gallery LLC, New York, NY. Reprinted with permission. Content Source credit: Photo of Virtual Helsinki by VR-Studio Zoan.

The results of Experiments 1A and 1B suggest that self-relevance is an important determinant of aesthetic appeal. However, because of the correlational nature of that analysis, it is difficult to isolate the influence of self-relevance. Thus, in Experiment 2, we sought to specifically manipulate the self-relevance of artworks in a controlled manner to test its influence on rated aesthetic appeal. To that end, we created self-relevant artworks with a deep convolutional neural network to implement style transfer (Fig. 2a). By using style transfer, the artistic style of an existing artwork was combined with the content of a photograph to create a new synthetic artwork with new content but the same style. For each participant, we thereby created custom artworks that were well controlled for both content and style, without having to explicitly ask participants about self-relevance. Novel artworks with personally relevant content were created by applying style transfer to images from online sources that reflected each participant’s unique responses to a questionnaire. This questionnaire asked about specific autobiographical memories, self-identity, interests, common activities, and preferences (Cultural Background and Lifestyle Questionnaire; see the Method section). For example, one participant wrote about a particularly memorable vacation to Helsinki; an image of a prominent central landmark in Helsinki was combined with a colorful artistic style to generate a novel artwork that reflected a memorable moment specific to that participant. In contrast, for a matched participant who expressed no particular link to Helsinki, the same artwork held no special relevance.

A total of 45 observers participated in the two-session online study. In the first session, the participants were asked a series of questions to identify self-relevant photographs. In the second session, they rated a set of artworks on several measures. In four blocks, the observers viewed 80 artworks and rated each of them on a single question. In both Block 1 (test) and Block 2 (retest), observers gave ratings for aesthetic appeal. In Block 3, observers rated the artworks for self-relevance. In Block 4, participants indicated whether the artwork was either definitely recognized, familiar, or unfamiliar (see the Method section). The artworks belonged to one of four conditions (Fig. 2b): (a) self-relevant artworks generated on the basis of their responses to the questionnaire; (b) other-relevant artworks that were generated for a matched participant; (c) a set of generated-control artworks shown to all participants; or (d) a selection of real artworks, also shown to all participants.

**Fig. 3.**
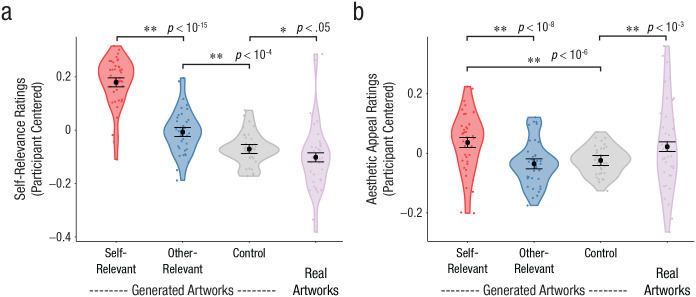
Effect of stimulus condition on ratings of (a) self-relevance and (b) aesthetic appeal in Experiment 2. Colored dots show average ratings for individual participants in each condition, after centering by participant, and shaded area indicates data density, smoothed with a gaussian kernel and trimmed. Black dots indicate the means, and error bars indicate 95% confidence intervals. Black horizontal brackets indicate significance. *N* = 40 participants.

We first validated our methodology by analyzing whether participants indeed rated the self-relevant artworks as more self-relevant. For this, we used an LMM with self-relevance modeled as a function of stimulus condition with participants as a random factor (Fig. 3a). Artworks from the self-relevant category were rated as significantly more self-relevant than all other categories (self-relevant vs. other-relevant estimate = 0.19), 95% CI = [0.16, 0.21], *d* = 0.59, *t*(3160) = 13.0, *p* = $1.3\;\times \;10^{-37}$ . Additionally, real artworks were rated as less self-relevant than the generated-control artworks (real-artworks vs. generated-control estimate = −0.031), 95% CI = [–0.059, –0.003], *d* = −0.097, *t*(3160) = −2.2, *p* = .031. Other-relevant artworks were also rated as more self-relevant than the generated-control artworks (other-relevant vs. control-generated estimate = 0.064), 95% CI = [0.036, 0.092], *d* = 0.20, *t*(3160) = 4.5, post hoc comparison adjusted *p* = $1.71\;\times \;10^{-5}$ using Holm method. This finding, that content that was selected as self-relevant for a given participant had a tendency to be more self-relevant for matched participants than the generated-control artworks, may reflect a degree of general shared experience across participants.

Next, we analyzed how aesthetic ratings differed between self- and other-relevant artworks (Fig. 3b). Artworks generated from self-relevant content were rated as significantly more appealing than matched other-relevant artworks (self-relevant vs. other-relevant estimate = 0.071), 95% CI = [0.048, 0.095], *d* = 0.27, *t*(3160) = 5.9, *p* = $3.8\;\times \;10^{-9}$ (LMM with aesthetic appeal modeled as a function of stimulus condition with participants as a random factor). In addition, although real artworks were rated as significantly more aesthetically appealing than the generated-control artworks (real-artworks vs. generated-control estimate = 0.046), 95% CI = [0.023, 0.070], *d* = 0.17, *t*(3160) = 3.8, *p* = .00013, the self-relevant-generated artworks recovered this difference, even being slightly preferred to the real artworks on average (self-relevant vs. real-artworks estimate = 0.014), 95% CI = [–0.0097, 0.038], *d* = 0.052, *t*(3160) = 1.2, *p* = .25 (not significant). Thus, generating new artworks with self-relevant content that related to a participant’s lived experience, identity, and interests was highly effective at increasing aesthetic appeal and did so in a manner that was independent of the artistic skill that characterized the real artworks.

We then examined how aesthetic appeal related to ratings of self-relevance at the level of individual artworks. To this end, we predicted participant ratings of aesthetic appeal from their ratings of self-relevance for all artworks from all four conditions. We found that the effect of self-relevance on aesthetic appeal was very strong (Fig. 1c): A change from not self-relevant to highly self-relevant increased aesthetic ratings by 35 points on a 100-point scale, slope = 0.35 for data rescaled to between 0 and 1, 95% CI = [0.32, 0.37], *t*(2940) = 25, *p* = $4.1\;\times \;10^{-122}$ . This finding further supports the conclusion that self-relevance is a major determinant of aesthetic appeal.

We wondered whether the effect of self-relevance might have been mediated by familiarity. To investigate this, we predicted aesthetic appeal from ratings of both self-relevance and familiarity (with random intercepts and slopes for participant and condition; see the Method section). We found that paintings that were definitely recognized or familiar were more preferred than unfamiliar paintings (Fig. 1d), slope = 0.086, 95% CI = [0.063, 0.11], *t*(2795) = 7.2, *p* = 7.9 × 10^-13^, and that definitely recognized paintings were slightly more preferred than merely familiar paintings, slope = 0.045, 95% CI = [0.010, 0.080], *t*(2855) = 2.5, *p* = .011. However, self-relevance was still a very strong predictor, slope = 0.30, 95% CI = [0.22, 0.37], *t*(18.9) = 8.1, *p* = $1.4\;\times \;10^{-7}$ , across all four image conditions (slopes of 0.31, 0.29, 0.26, and 0.33 for self-relevant, other relevant, generated-control, and real artworks, respectively). A formal mediation analysis revealed that familiarity was able to explain only a small fraction of the self-relevance effect (Fig. 1e; average causal mediation effect = 0.04, 95% CI = [0.03, 0.05]; remaining direct effect of self-relevance on aesthetic appeal = 0.33, 95% CI = [0.30, 0.35]). Thus, we conclude that the effect of self-relevance was not mediated by familiarity.

### Artworks reflecting one’s self-construct were more appealing

**Fig. 4.**
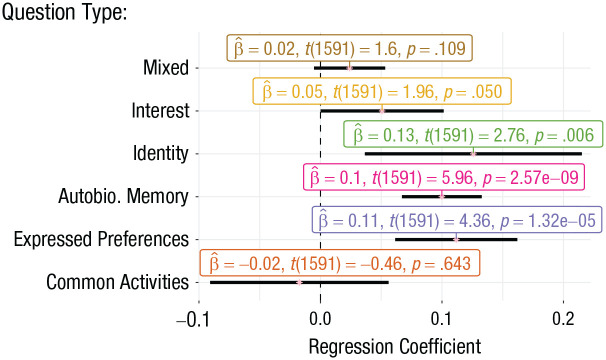
Artworks reflecting one’s self-construct were more appealing. Artworks in the self-relevant condition that reflected specific autobiographical (Autobio.) memories (238 items), identity (26 items), expressed preferences (87 items), and interests (86 items) were rated as significantly more aesthetically appealing than generated-control artworks (baseline, 800 items), whereas artworks reflecting common activities (39 items) or those derived from questions reflecting “mixed” (324 items) aspects of self-relevance were not. Black bars indicate 95% confidence intervals. *N* = 40 participants.

A stimulus might be seen as relevant for different reasons, such as relating to a specific autobiographical memory or an aspect of one’s self-identity or being relevant for one’s current goals (Priniski et al., 2018). We therefore sought to determine which aspects of self-relevance influenced aesthetic appeal. To this end, we reclassified the self-relevant synthetic artworks into five subclasses on the basis of the nature of the questionnaire item from which they were derived: (a) specific autobiographical memories, (b) interests, (c) aspects of personal identity, (d) common activities, and (e) explicitly expressed preferences. In addition, we identified a sixth mixed class. Ratings of aesthetic appeal for the 20 participant-specific generated artworks in the self-relevant condition were modeled as a function of these six classes (see the Method section). Artwork classes that were most associated with higher aesthetic ratings (Fig. 4) were autobiographical memories, slope = 0.10, 95% CI = [0.07, 0.13], *t*(1555) = 6.0, *p* = $3.2\;\times \;10^{-9}$ ; identity, slope = 0.13, 95% CI = [0.04, 0.21], *t*(1558) = 2.8, *p* = .0058; expressed preferences, slope = 0.11, 95% CI = [0.06, 0.16], *t*(1556) = 4.4, *p* = $1.4\;\times \;10^{-5}$ ; and interests, slope = 0.051, 95% CI = [0.0002, 0.10], *t*(1557) = 2.0, *p* = .050. Artworks that related to common activities, however, were not associated with higher aesthetic ratings, slope = −0.017, 95% CI = [–0.09, 0.06], *t*(1558) = −0.46, *p* = .64, nor were artworks in the mixed class, slope = 0.024, 95% CI = [–0.005, 0.05], *t*(1555) = 1.6, *p* = .11. This analysis confirms that artworks that related to aspects of an individual’s self-construct, such as specific autobiographical memories and identity, were rated as most appealing. The lack of effect for common activities suggests that goal relevance may not be sufficient to increase aesthetic appeal.

From the above analyses, we conclude that the primary effect on aesthetic appeal is driven by subjective aspects of observers’ responses to the paintings. To determine the degree to which the perceptual effects of the style-transfer procedure may have influenced aesthetic ratings, we computed a measure of perceptual similarity between each generated artwork and its source image on the basis of network activation in a VGG neural network for object recognition (LPIPS; see Uran et al., 2022). This allowed for an assessment of whether the “content loss” of the synthetic artworks (e.g., the degree to which the original photographic content was no longer accessible) was related to their aesthetic appeal. On average, there was no difference in average content loss between self-relevant and other-relevant conditions (self-relevant μ=0.564 , σ=0.033 ; other-relevant μ=0.564 , σ=0.040 ), *d* = 0.0084, two-tailed paired-samples *t*(39) = 0.033, *p* = .97. In addition, LPIPS scores were not correlated with trial-wise aesthetic ratings (for self-relevant trials, *r* = −.046, *p* = .19; for other-relevant trials, *r* = .00023, *p* = .99). Hence, the degree to which the style-transfer process rendered the novel artworks as perceptually dissimilar from the original photographs was not predictive of aesthetic appeal.

## Discussion

Experiences with artwork are paradigmatic examples of how interactions with the external world reach inside and affect a person. Such experiences tap into personally lived experience, and when directly measured, aesthetic tastes in visual artwork are highly individual (Leder et al., 2016; Vessel et al., 2013, 2018). We found that aesthetic ratings of visual art are strongly correlated with self-relevance judgments (Experiments 1A and 1B). Only a small portion of the self-relevance effect could be related to intrinsic image properties. Directly manipulating the self-relevance of artworks strongly affected their rated appeal: Individually customized synthetic artworks generated on the basis of participants’ responses to a Cultural Background and Lifestyle Questionnaire were rated as more aesthetically appealing than either artworks generated for a different participant or a control set of artworks shown to all participants (Experiment 2). Explicit self-relevance ratings, collected after the aesthetic ratings, confirmed that participants found customized artworks to be highly self-relevant, indicating successful manipulation of self-relevance.

Why is content related to one’s self-construct more likely to be experienced as aesthetically appealing? An answer may lie in the link between aesthetic appeal and information gain: High-order semantic and associative information generally matters more than low-level perceptual features for determining the aesthetic value that a person assigns to a visual image or experience (Palmer & Schloss, 2010; Schepman & Rodway, 2019; Vessel & Rubin, 2010). Particularly in the context of a predictive-processing account of the mind/brain (Clark, 2013; Rao & Ballard, 1999), this is consistent with theories that posit that sensemaking and uncertainty reduction are positively valued and experienced as pleasurable (Biederman & Vessel, 2006; Schmidhuber, 2010). Importantly, our knowledge about the world is structured hierarchically, and information gains that relate to higher nodes of a knowledge hierarchy (e.g., resolution of conceptual ambiguity or a change in beliefs) allow for a greater reduction in overall uncertainty about our model of the world. Supporting evidence for this account has been reported in both the auditory (Cheung et al., 2019; Sarasso et al., 2021) and visual (Van de Cruys et al., 2021) domains.

Information about the self resides at the top of that knowledge structure, and research in social psychology suggests that it matters more. In particular, work exploring the mnemonic advantage for self-referential encoding (the self-reference effect; Klein & Loftus, 1988; Rogers et al., 1977) shows that the self-construct is “special”; it is organized differently and relies on unique mental and neural processes (Axelrod et al., 2017; Moran et al., 2009; Spreng & Grady, 2010) in a way that reference to the self confers an encoding advantage. Part of this specialness relates to its unique position at the center of our structured knowledge about the world, which promotes deeper processing (Gillihan & Farah, 2005; Wagner et al., 2012). Given the centrality of the self-construct, it follows that acquiring information that relates to the self, and hence has the capacity to reduce uncertainty about central aspects of our world model, leads to greater “aha,” greater pleasure, and higher aesthetic valuation than a change in beliefs or resolution of ambiguity about a nonpersonal object or about a resolution of a perceptual ambiguity.

Yet self-relevance is not the only way that artworks can acquire aesthetic value. Real artworks were rated low on self-relevance but as more aesthetically appealing than generated-control artworks in Experiment 2, indicating the presence of something in real artworks that generated artworks did not capture. The high aesthetic appeal of real artworks may be attributable to the artistic skill reflected in these works, although we cannot rule out a potential role for the larger range of styles, content, and abstraction found in the set of real artworks. In comparison, the fact that generated artworks with self-relevant content resulted in similarly high ratings of aesthetic appeal suggests that the effect of self-relevance on aesthetic value is largely independent of factors related to perceived artistic skill. Input styles were matched across self-relevant and other-relevant conditions, ruling out any contribution of residual artistic differences in styles to the observed difference in rated appeal for generated self-relevant artworks.

The effect of self-relevance on aesthetic appeal for artwork was largely independent of specific image features. In Experiment 2, each participant’s self-relevant images became other-relevant images for a matched participant. Thus, there were, on average, no stimulus differences between self-relevant and other-relevant conditions. In Experiment 1A, aesthetic appeal and the effect of self-relevance on aesthetic appeal were largely independent of a model of image features. This agrees with the low estimates of shared self-relevance and shared aesthetic appeal, which define a ceiling for how much variance can be accounted for by image properties. In contrast, shared factors account for much more variance for natural kinds (Vessel et al., 2018). Indeed, a significant portion of variance in scene preferences can be predicted from naturalness and disorder (Kotabe et al., 2017). The memorability of both scenes and faces, although apparently unrelated to aesthetic value, is also strongly predictable from image features (Needell & Bainbridge, 2022). For the artworks used here, the variance in aesthetic appeal accounted for by self-relevance was much higher than the combined effect of 18 image features (28% vs. 8%). Only a small portion of the relationship between self-relevance and aesthetic appeal could be related to the image feature model (5%, or ≈1/5 of the total effect). The majority of the effect of self-relevance was at an individual level. Further research is needed to determine how self-relevance and image features interact when in conflict.

Information about familiar concepts may also be processed more deeply (Craik & Lockhart, 1972). Yet whereas rated familiarity positively predicted aesthetic ratings in Experiment 2, the effect of self-relevance on aesthetic appeal was not mediated by familiarity. The biggest effect came from the contrast of familiar versus unfamiliar artworks, but explicit recognition of content (vs. being merely familiar) also predicted higher aesthetic appeal with a smaller effect. This finding agrees with studies showing that previous exposure increases liking (the “mere exposure” effect; Park et al., 2010; Zajonc, 1968) and the association of perceptual and conceptual fluency with greater liking (Reber et al., 2004). However, cases exist in which observers show preferences for novel stimuli (Biederman & Vessel, 2006; Park et al., 2010); for example, we found that real artworks were rated as less familiar than self-relevant-generated artworks but received high aesthetic ratings. Despite the influence of familiarity, we found that the presence of self-related content led to higher aesthetic appeal even when the artwork’s content was not specifically rated as familiar (Fig. 1e, mediation analysis).

We also observed higher aesthetic ratings for generated artworks whose content reflected explicitly expressed preferences. Although such preferences can be considered a part of one’s self-construct, in the context of the current study, it was important to separate out their contribution to avoid circularity. By doing so, it became clear that reference to aspects of the self-construct that were not simply expressed preferences also led to robust increases in appeal. However, generated artworks reflecting common activities were not rated as more appealing, although only very few items (39) contributed to this category, making it difficult to assess its reliability. Further work is needed to assess whether reference to such common activities, which do not necessarily reflect the self-construct but may instead reflect momentary goal relevance, affects aesthetic appeal.

Artworks in the other-relevant condition were rated as more self-relevant than generated-control artworks. This difference likely reflected general shared experience across participants, who were all German-speaking individuals living in Europe. Importantly, all of the other-relevant artworks were created for specific individuals, whereas the generated-control stimuli were not. Generated-control artworks therefore depicted objects or concepts that were less likely part of any person’s self-construct (e.g., nondescript objects or places). Similarly, higher self-relevance ratings for generated artworks compared with real artworks likely reflected the presence of fewer references to specific objects in the preselected artworks, compared with stimuli created from photographs. Although the observed effects were very robust across individual participants, future work is needed to test whether these findings generalize to more culturally and demographically diverse populations.

Experiences with art can be transformative. Yet even for mundane circumstances, aesthetic factors can strongly influence behavior, mood, and productivity. Our results show a tight connection between aesthetic appeal and one’s sense of self. We note that people do not always engage with artwork to seek an experience of “self”; often, artwork serves as a bridge to experiencing and understanding the “other.” Yet when an art experience relates to one’s self-schema, that knowledge structure can act as a key to unlock deeper processing, greater understanding, and more pleasure. Beyond aesthetic experiences, we predict that a similarly tight relationship would be observed for other forms of pleasure from comprehension (e.g., insight problem solving and creative inspiration; Welke et al., 2023). These results bear on an increasingly urgent problem in our digitally immersive world. Recommender systems designed to deliver personalized content (e.g., TikTok, Instagram) have become ubiquitous, and psychologists have flagged their potential for problematic use (e.g., Su et al., 2021). Understanding the link between self-relevance and aesthetic engagement is critical for developing guidelines for safe use.

Contrary to the commonly held view that beauty primarily exists as an objective property of the world that humans can perceive, we showed that an artwork’s aesthetic value also depends strongly on its resonance with the self-construct, a core and highly individual aspect of structured knowledge. For many people, aesthetic tastes are central to their identity (Fingerhut et al., 2021), and at least for artwork, resonance with one’s lived experience is of central importance for aesthetic appeal.

## Supplemental Material

sj-pdf-1-pss-10.1177_09567976231188107 – Supplemental material for Self-Relevance Predicts the Aesthetic Appeal of Real and Synthetic Artworks Generated via Neural Style Transfer Supplemental material, sj-pdf-1-pss-10.1177_09567976231188107 for Self-Relevance Predicts the Aesthetic Appeal of Real and Synthetic Artworks Generated via Neural Style Transfer by Edward A. Vessel, Laura Pasqualette, Cem Uran, Sarah Koldehoff, Giacomo Bignardi and Martin Vinck in Psychological Science

## Transparency

*Action Editor:* Vladimir Sloutsky

*Editor:* Patricia J. Bauer

*Author Contribution(s)*

**Edward A. Vessel:** Conceptualization; Formal analysis; Funding acquisition; Investigation; Methodology; Project administration; Supervision; Visualization; Writing – original draft; Writing – review & editing.

**Laura Pasqualette:** Conceptualization; Data curation; Formal analysis; Investigation; Methodology; Project administration; Writing – original draft; Writing – review & editing.

**Cem Uran:** Formal analysis; Methodology; Software; Writing – review & editing.

**Sarah Koldehoff:** Conceptualization; Data curation; Formal analysis; Investigation; Methodology; Project administration; Writing – review & editing.

**Giacomo Bignardi:** Formal analysis; Software; Visualization; Writing – review & editing.

**Martin Vinck:** Methodology; Writing – review & editing.

**Open Practices:** The studies reported in this article were not preregistered. Deidentified data and code for all studies are publicly accessible at https://osf.io/6zxc5. Executable code and tutorial to reproduce Variance Component Analysis as carried out in this study are available at https://github.com/giacomobignardi/empirical-aesthetics-VCA/tree/main. Generated artworks are also available. Real artworks are not included as they contain images for which the authors do not have distribution rights. Please direct requests for access to the corresponding author.
