# Supplementary material for: Self-Relevance Predicts the Aesthetic Appeal of Real and Synthetic Artworks Generated via Neural Style Transfer
Source: Psychol Sci. 2023 Aug 14;34(9):1007–23. doi: 10.1177/09567976231188107 (PMC7616853; doi:10.1177/09567976231188107)
Supplement: sj-pdf-1-pss-10.1177_09567976231188107 – Supplemental material for Self-Relevance Predicts the Aesthetic Appeal of Real and Synthetic Artworks Generated via Neural Style Transfer [file sj-pdf-1-pss-10.1177_09567976231188107.pdf]

Supplementary Materials

Vessel, E.A., Pasqualette, L.B., Uran, C., Koldehoff, S., Bignardi, G., Vinck, M. (2023) Self-relevance predicts the aesthetic appeal of real and synthetic artworks generated via neural style transfer. *Psychological Science.* https://doi.org/10.1177/09567976231188107

Comparison of Self-Relevance to a Model of Intrinsic Image Properties

For Experiment 1A, we compared how well ratings of self-relevance were able to predict aesthetic appeal to a model of intrinsic image properties. A set of 15 features were derived from each image, along with scores for memorability (Needell and Bainbridge, 2022) and average ratings of naturalness and disorder (Kotabe et al., 2017) from an independent set of raters (see Methods).

Image Feature Model

A model comprising a set of low- and mid-level features was created in order to test the degree to which the effect of self-relevance on aesthetic appeal could be mediated by specific image features.

Predictability for a given image was computed as the average predictability across 16 non-overlapping sub-regions of the whole image. For every sub-region predictability was computed comparing what is in the region to the prediction of an inpainting algorithm as described in (Uran et al., 2022).

Compressibility was computed as the negative bits per pixel (bpp) which is commonly used to benchmark image compression methods. For image compression, we used a context-adaptive, entropy-based deep neural network model (Lee et al., 2018).

Visual Saliency was computed using SalGAN, a state-of-the art generative adversarial network trained for visual saliency prediction of the image (Pan et al., 2017; Wagatsuma et al., 2021). Visual saliency was quantified as the average predicted value over the image.

HSV Hue, Saturation, Value were computed using rgb2hsv function of Matlab.

Root-mean-square(RMS) Contrast was computed as the standard deviation of the image.

Fourier Slope, Fourier Sigma (Deviation), Self-Similarity, Complexity, Anisotropy were computed as described in (Geller et al., 2022; Redies et al., 2020).

Dimensionality was determined by taking the rotational average of the Fourier spectrum, and ranking the spectral components by magnitude. Dimensionality was then defined as the slope of the resulting spectrum (Uran et al., 2022).

Spectral Centroid is the center of mass of the power spectrum, defined as the mean spatial frequency in the image, weighted by the total power in the frequency bands.

Spectral Variance is the circular variance of the power spectrum.

Memorability was computed using the ResMem network (Needell and Bainbridge, 2022).

In addition to the features computed from the images, we collected ratings of naturalness and disorder from a new set of observers (Kotabe et al., 2017). 27 observers were recruited via Prolific and participated in an experiment coded using the OpenSesame experiment builder ((Mathôt et al., 2012), https://osdoc.cogsci.nl), hosted on the Max Planck Institute for Empirical Aesthetics (MPIEA) JATOS server (https://www.jatos.org). Every participant rated all 148 images; 6 images were repeated in order to compute a test-retest reliability score. On each trial, the image was presented for 5 seconds, followed by a screen on which they were required to rate the image for both naturalness and disorder (question order reversed for half the participants). Participants responded by using the mouse to click on a location on a continuous slider bar for each question. A smaller version of the image was presented next to the rating scales. Once both questions had been answered, the participant clicked a “next” button to proceed to the next trial.

A test-retest score was computed for both questions by calculating the Pearson correlation between the first and second set of ratings for the 6 repeated images. As no participant’s test-retest score was below 0.5 for both naturalness and disorder, no participants were removed. Average naturalness and disorder scores were then computed for each image by computing the mean rating across all 27 participants.

Analysis and Results

First, we individually fit each of the following linear mixed models using *lmer* and compared the self-relevance slope coefficients to the base model with self-relevance as the only fixed effect:

$AesthRatings \sim SelfRelevRatings + Naturalness + Disorder + (SelfRelevRatings|Participant) + (1|Image)$

$AesthRatings \sim SelfRelevRatings + Memorability + (SelfRelevRatings|Participant) + (1|Image)$

$AesthRatings \sim SelfRelevRatings + HSV_h + HSV_s + HSV_v + FourierSigma + fourierSlope + SelfSimilarity + Complexity + Anisotropy + Compressibility + Dimensionality + Predictability + VisualSaliency + RMSContrast + SpectralCentroid + SpectralVariance + (SelfRelevRatings|Participant) + (1|Image)$

When compared to the base model, all changes in the slope coefficient for self-relevance were smaller than 0.01 (all slopes = 0.36, t[32.3] = 12.3, p =< .001). Results were virtually unchanged when fitting a model inclusive of all 18 image property variables, resulting in a trivial drop of .01 (from slope 0.36 to slope 0.35, see Table S1 and Figure S1b).

Table S1: Slope coefficients for a set of models comparing self-relevance to a set of intrinsic image properties. Second value is the standard error. Significance codes: *** < 0.001, ** < 0.01, * < 0.05, . < 0.1. *HSV* hue saturation value model, *S.F.* spatial frequency, *Var.* variance.

Predictor	Base Model	Base+ND	Base+M	Base+Vis	Base+All
(Intercept)	0.32±0.02***	0.14±0.05**	0.48±0.05***	0.47±0.11***	0.48±0.14**
Self Relevance	0.36±0.03***	0.36±0.03***	0.36±0.03***	0.36±0.03***	0.36±0.03***
Naturalness		0.22±0.05***			0.07±0.06
Disorder		0.15±0.06*			0.06±0.08
Memorability			−0.22±0.05***		−0.16±0.07*
HSV Hue				0.05±0.03	0.05±0.03.
HSV Saturation				−0.03±0.04	−0.01±0.04
HSV Value				−0.09±0.04*	−0.07±0.04*
Fourier Sigma				−0.02±0.05	−0.06±0.05
Fourier Slope				0.01±0.06	−0.04±0.06
Self Similarity				−0.02±0.06	0.01±0.06
Complexity				0.03±0.09	0.04±0.08
Anisotropy				−0.11±0.05*	−0.07±0.05
Compressibility				−0.01±0.11	0.06±0.11
Dimensionality				−0.05±0.07	−0.07±0.07
Predictability				0.04±0.05	0.03±0.05
Visual Saliency				−0.14±0.04***	−0.15±0.04***
RMS Contrast				0.01±0.06	0.03±0.05
S.F. Centroid				−0.08±0.06	−0.06±0.07
S.F. Circular Var.				0.00±0.03	0.01±0.03

Second, we performed a variance decomposition analysis to directly compare how much variance in aesthetic appeal could be accounted for by self-relevance and by the image feature model. To do so, we adapted the Variance Component Analysis (VCA) described in the Methods to include fixed-effect terms for self-relevance and for the image feature model. To simplify their interpretation, we did not include the Block term and its interactions with the other random effects. Each of the following models were fit using *lmer*. ImageFactors refers to the full set of 18 image property variables.

Null model:
$AesthRatings \sim 1 + (1|particip) + (1|stim) + (1|particip : stim)$

Self-Relevance (SR) model:
$AesthRatings \sim selfRelevRatings + (1|particip) + (1|stim) + (1|particip : stim)$

Image Factors (IF) model:
$AesthRatings \sim ImageFactors + (1|particip) + (1|stim) + (1|particip : stim)$

Self-Relevance plus Image Factors (SR+IF) model:
$AesthRatings \sim SelfRelevRatings + ImageFactors + (1|particip) + (1|stim) + (1|particip : stim)$

For the null model we computed the Variance Partitioning Coefficients (VPC) as the proportion of the random effect term's variance over the total variance. For each of the models including at least one fixed effect, VPCs were calculated as the proportion of the random effect term's variance for that model over the total variance of the null model. By comparing the relative drop in VPC of these models, we were able to partition the variance in ratings of aesthetic appeal into VPCs related to the participant, stimulus, participant by stimulus interaction, and residual variance, and track how these values changed when adding in self-relevance and the image feature model (Fig. S1c).

As reported in the main results, the image feature model was able to account for $\approx 8\%$ of the variance in ratings of aesthetic appeal; as the image feature model is the same for all participants, this variance is confined to the stimulus-related variance ($\approx 15\%$; Fig. S1c, between the dashed and solid lines). Self-relevance was able to predict a total of $\approx 28\%$ of the variance (Fig. S1d), of which $\approx 7\%$ was related to the stimulus component, $\approx 5\%$ to the participant component, and $\approx 16\%$ to the stimulus by participant interaction component. The overlap between self-relevance and the image feature model was $\approx 5\%$ (Stimulus_SRIF). An additional $\approx 5\%$ of the stimulus-related variance was not explained by either the image feature model nor self-relevance.

Finally, we performed a dominance analysis (Azen and Budescu, 2003) of the relative contribution of self-relevance and image features to aesthetic appeal, using the *domin* function from the *domir* package in R (Luchman, 2022). This analysis was only possible using ratings that were averaged across individuals, and was therefore restricted to describing the effect of shared self-relevance on shared taste, compared to image features. The results clearly indicate that average self-relevance contributed more than any image feature to the average aesthetic appeal of an image. The average relative increase of the fit statistics (R^2) when adding self-relevance to the combination of all possible 524287 ($2^{Npredictors} - 1$) multiple linear models ranked first amongst all the predictors, (general dominance $D = 0.29$, Standardized General Dominance, $D = 0.38$). Naturalness was the second-most dominant factor (general dominance $D = 0.10$), followed by Visual Saliency (general dominance $D = 0.06$), Memorability (general dominance $D = 0.05$) and Disorder (general dominance $D = 0.04$).

We note that the aim of the analyses in this section was not to derive a complete model, but rather to estimate the degree to which the effect of self-relevance on aesthetic appeal was independent of intrinsic image properties. We would therefore urge caution in interpreting the results beyond this purpose. Moreover, we note that the estimates for the amount of variance accounted for by the introduction of the fixed effects might vary in their degree of precision, and they are thus only approximate estimates. This is due to the fact that for each model, estimates for the variances unaccounted for by the fixed effect can be slightly higher than the ones in the null model (in our analysis, up to a .02 increase for the residuals and up to $=<$ .001 for other VPC), which results in small negative differences between VPCs. The presence of negative differences might upwardly bias, in total, estimates to the maximum of the sum of the total negative differences, which in our case was -.02.

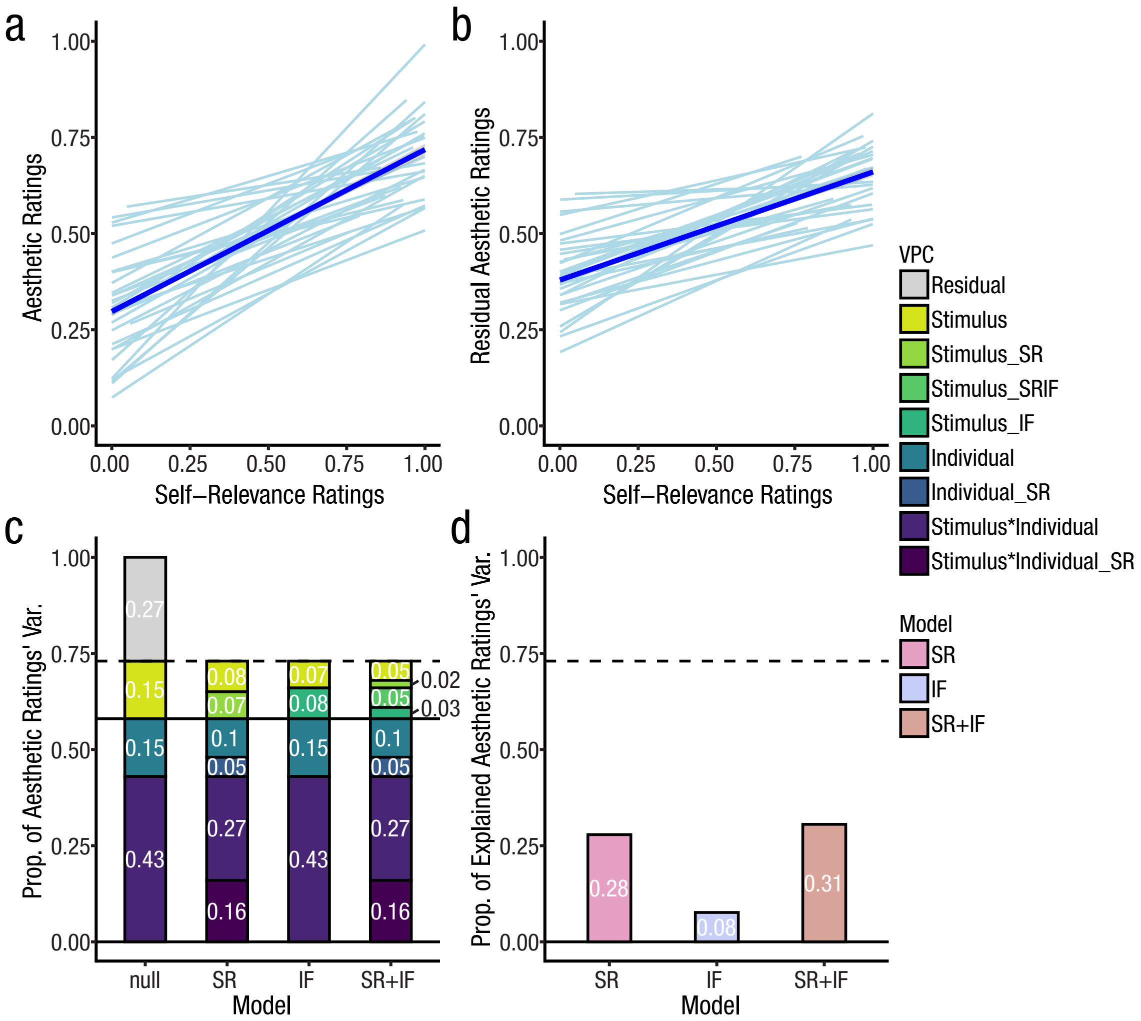

Figure S1: Impact of image properties on the relationship between self-relevance and aesthetic appeal. a) Linear fit for prediction of ratings of aesthetic appeal from self-relevance for Exp. 1A, replotted from Fig. 1a for comparison. b) This relationship between self-relevance ratings and aesthetic appeal remains even after accounting for the full image feature model, with an average slope of 0.35. For both plots, the thick dark blue line indicates the average slope (with the standard error of the estimated slope in light gray), and the thin, light blue lines show the fits for individual participants. Residual Aesthetic Ratings refer to the residuals after regressing the fit for the image feature model from ratings for aesthetic appeal. c) Aesthetic ratings Variance Partitioning Coefficients (VPC, color coded) obtained via Variance Component Analysis (VCA) (Martinez et al., 2020; Sutherland et al., 2020). Each VPC is expressed as the random effect variance over the total variance obtained from the null model. VPC for fixed effects are obtained as the differences between VPCs across models. Each bar represents the VPC for the respective models. Note that the total repeatable variance estimated from this null linear mixed model is 72%, which is 3% lower than the one reported in the main text (repeatable variance 75%). This is a consequence of the omission of the Block (exposure) term as a random effect, which now contributes to the residual variance. We note that in the extended null model reported in the main text, we found 3% of the total variance to be accounted for by the subject by exposure interaction, accounting for the difference observed in this model. d) The approximate proportion of the total variance explained by each model is computed as the sum of the difference between the VPCs obtained from the null and the model with the respective fixed effect(s).

Exp. 1B: ”Being Moved”

In Experiment 1B, participants rated each image using separate sliders for how much they got the feeling of beauty, and also for how moved they were by each image of artwork. Linear fits for the ”beauty” question are shown in Fig.

1b. Fits for the "being moved" question are shown here (Fig. S2). See below (Full Instructions) for the wording of each individual question.

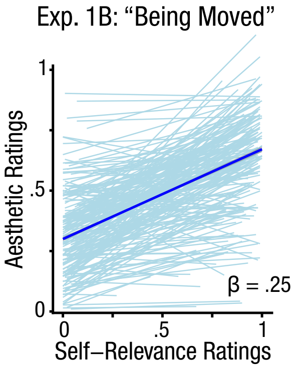

Figure S2: In Exp. 1B, self-relevance predicted ratings of "being moved" with an average slope of 0.25 (N=208). Thick dark blue line indicates the average linear slope (with the standard error of the estimated slope in gray) and the thin light blue lines show linear slopes for individual participants.

Exp. 1B: Reanalysis Including Low-Reliability Participants

The data from Exp. 1B was reanalyzed after including all participants regardless of their test-retest score, minus those that failed the attention check (N=234). The reported slopes changed only very slightly, both for self-relevance predicting beauty (slope = 0.30, 95% CI [0.27 0.33], t[147] = 20.0, $p = 7.3 \times 10^{-44}$, partial $\eta^2 = 0.73$), and for self-relevance predicting being moved (slope = 0.24, 95% CI [0.21 0.27], t[146] = 15.0, $p = 2.5 \times 10^{-31}$, partial $\eta^2 = 0.61$). This suggests that the results are robust and not a consequence of removing low-reliability participants.

Nonlinear Fits

Multilevel linear fits (with participant-specific slopes) of the relationship between aesthetic appeal and self-relevance are reported in the results and Fig. 1. Here (Fig. S3), we include plots of all trials, fitted with a local nonlinear fit in order to more clearly illustrate the relationship between aesthetic appeal and self-relevance. The relationship is very consistent across 3 experiments and variations on dependent measures of aesthetic appeal: while aesthetic appeal monotonically increases across the full range of self-relevance ratings, the slope of the relationship is steeper near extreme values of self-relevance. This may be in part a reflection of the non-uniform distribution of self-relevance ratings.

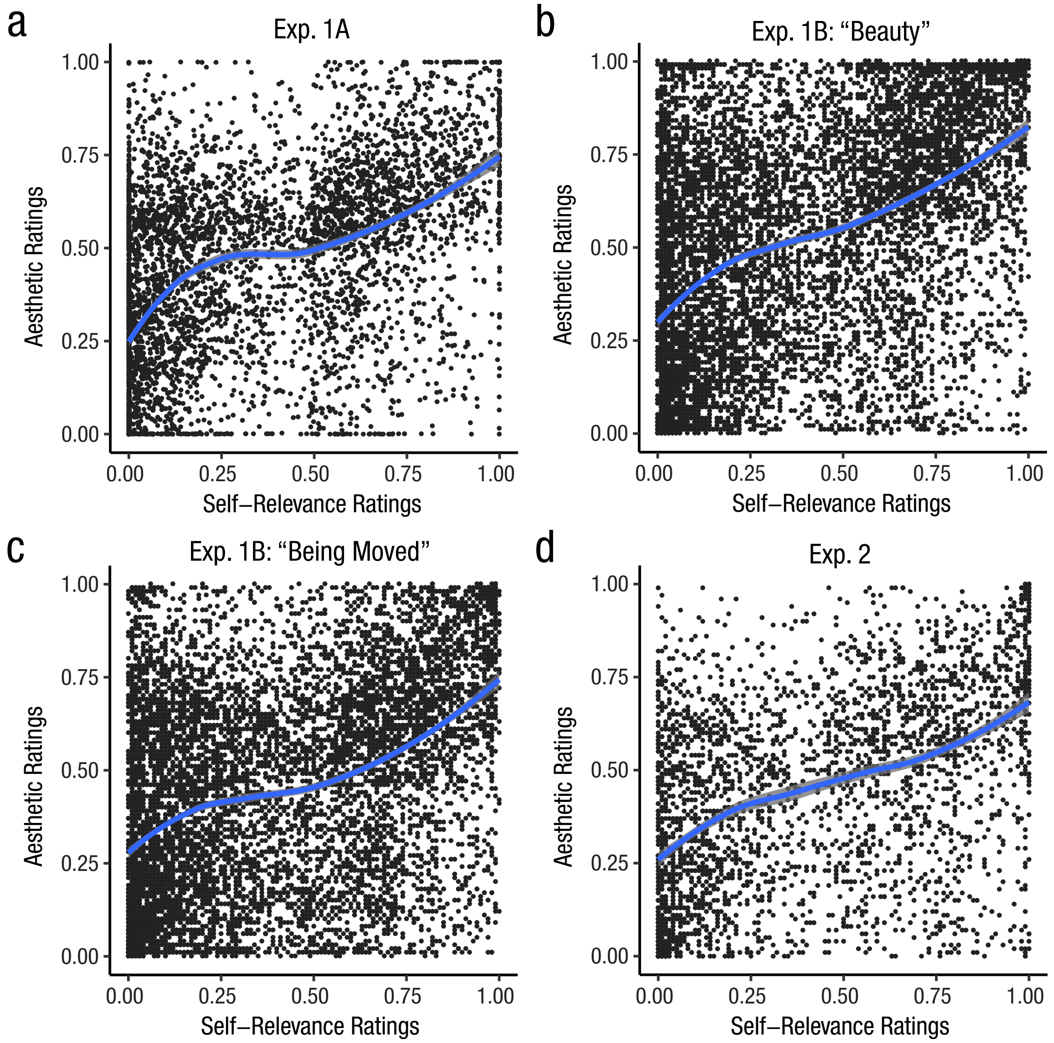

Figure S3: Nonlinear fits of the relationship between aesthetic appeal and self-relevance. a) Scatterplot for Experiment 1A of the first round of aesthetic ratings (Block 1) versus the first round of self-relevance ratings (Block 2); N = 32 participants, rating 148 artworks. b) Scatterplot for Experiment 1B of ratings of ”beauty” (Block 1) versus self-relevance ratings (Block 2); N = 208 participants, rating 42 artworks. c) Scatterplot for Experiment 1B of ratings of ”being moved” (Block 1) versus self-relevance ratings (Block 2); N = 208 participants, rating 42 artworks. d) Scatterplot for Experiment 2 of the first round of ratings of aesthetic appeal (Block 1) versus self-relevance ratings (Block 3); N = 40 participants rating 80 artworks. Blue lines indicate local fits of a 2nd-degree polynomial using the *loess* function in R with default smoothing parameter $\alpha = 0.75$; gray band is the standard error of this fit.

Exp. 2 Test-Retest Reliability and Repeatable Variance

In Exp. 2, each observer viewed and rated each artwork for aesthetic appeal twice (in separate blocks) before making ratings of self-relevance (Block 3) and judgments of familiarity (Block 4). A Pearson correlation was computed between the first and second block for each participant to derive a test-retest reliability score. The average reliability score of all 45 participants was 0.775. As reported in the Methods, 5 of the participants had reliability scores below 0.5 and were therefore excluded from further analysis. The average reliability score of the remaining participants was 0.802. By squaring the individual reliability scores and then averaging, we obtain an estimate of the amount of repeatable variance in the aesthetic rating data of 62.3%. We use this method due to the fact that different participants saw different stimuli; the variance partitioning method used in Exp. 1A was not possible for this dataset.

List of Artworks

1A: used as a stimulus in Experiment 1A; 1A*: also used in the retest block of Experiment 1A; 1B: used as a stimulus in Experiment 1B; 1B*: also used as a retest image in Experiment 1B; 2R: used as a real artwork in Experiment 2; 2S: used as a source image in Experiment 2.

. . . The Foggy Foggy Dew, 1968-1970, Jochen Seidel (1924-1971, German). Mixed media on canvas. 1A* 2S

8 Vinegar Bottles, 1972-1973, Janet Fish (1938-, USA). Oil on canvas. 1A* 1B

A Passing Storm, 1849, Frederic Edwin Church (1826-1900, USA). Oil on canvas. 1A 2S

A Street in Venice, 1880-1882, John Singer Sargent (1856-1925, USA). Oil on canvas. 1A* 1B

A Windmill on a Polder Waterway, Known as 'In the Month of July', ca. 1889, Paul Joseph Constantin Gabriël (1828-1903, Dutch). Oil on canvas. 2S

After Leslie Left, 1983-1984, Janet Fish (1938, USA). Oil on canvas. 2S

Akrura's Vision of Vishnu/Krishna, c. 1760-1765, unknown, Kangra, Himichal Pradesh, India (unknown, Pradesh). Opaque watercolor with gold on paper. 1A

An Actress (Portrait of Suzanne Santje), 1903, Thomas Eakins (1844-1916, USA). Oil on canvas. 1A

An Ecclesiastic, 1874, Mariano Fortuny y Marsal (1838-1874, Spain). Oil on panel. 1A*

Angel with Crown, 19th c. , unknown, Iran (unknown, Iran). Oil on canvas. 1A

Apple Bloom, 1903, Robert Vonnoh (1858-1933, USA). Oil on canvas. 1A

Apples in a Dish, 1883, Pierre-Auguste Renoir (1841-1919, France). Oil on canvas. 2R

Approaching Storm Beach Near Newport, 1861-62, Martin Johnson Heade (1819-1904, USA). Oil on canvas. 1A

Autumn Forest Interior, c. 1860, Narcisse Virgile Diaz de la Peña (1807-1876, France). Oil on panel. 1A 2R

Bather with a cigarette, 1924, Yasuo Kuniyoshi (1889-1953, USA). Oil on canvas. 1A

Beloved Name, c. 1865-1918, Luigi Nono (1860-1918, Italy). Oil on canvas. 1A 2R

Birds Gather under the Spring Willow, late 1400s-early 1500, Yin Hong (ca. 1500, China). Hanging scroll, ink and color on silk. 2R

Blonde Boy with Primer, Peach, and Dog, 1836, Ammi Phillips (1788-1865, USA). Oil on canvas. 1A

Bouquet of Flowers in a Faience Vase, c. 1600-1620, Jan Brueghel the Younger (1601-1678, Flemish). Oil on wood. 1A 2S

Bouquet of Violets in a Vase, 1882, Henri de Toulouse-Lautrec (1864-1901, France). Oil on panel. 1A

Breton Women Attending a Pardon, 1892, Emile Bernard (1868-1941, France). Oil on cardboard. 2S

Camp Butler, Maryland, ca. 1861, Unknown (?, USA). Oil on panel. 2R

Capture of H. B. M. Frigate Macedonian by U. S. Frigate United States, October 25, 1812, 1852, Thomas Chambers (1808-1879, USA). Oil on canvas. 1A

Celia Thaxter's Garden, Isles of Shoals, Maine, 1890, Childe Hassam (1859-1935, USA). Oil on canvas. 2R

Celtic Tale, 1894, Paul Serusier (1863-1927, France). Oil on canvas. 1A 2S

Child in Rocking Chair, 1870, E. L. George (19th century, USA). Oil on canvas. 1A

Chiron Instructing Achilles in the Bow, c. 1776, Giovanni Battista Cipriani (1727-1785, Italy). Oil on canvas. 1A

Christ on the Sea of Galilee, 1853, Ferdinand-Victor-Eugene Delacroix (1798-1863, France). Oil on composition board. 1A 1B 2S

Chrysanthemus, 1874-1876, James Tissot (1836-1902, France). Oil on canvas. 1A 1B

Classical Landscape, c. 1830, Joshua Shaw (1777-1869, USA). Oil on canvas. 1A 2S

Clearing Up Coast of Sicily, 1847, Andreas Achenbach (1815-1910, Germany). Oil on canvas. 1A 2S

Cloud Study, c. 1822, John Constable (1776-1837, Britain). Oil on paper. 1A 2S

Cloud Study, c. 1822, John Constable (1776-1837, Britain). Oil on paper. 1A 2R

Connecticut Barns in Landscape, 1934, Charles Sheeler (1883-1965, USA). Oil on canvas. 2R

Constant, 1988, Valerie Jaudon (1945-, USA). Oil on canvas. 1A 1B

Cottage on Fire, c. 1786-1787, Joseph Wright (1734-1797, Britain). Oil on canvas. 1A 1B 2S

Cyclamen, 1918-19, Ernst Ludwig Kirchner (1880-1938, Germany). Oil on canvas. 2S

Dance Hall Bellevue [obverse], ca. 1909-1910, Ernst Ludwig Kirchner (1880-1938, Germany). Oil on canvas. 2S

Death on the Pale Horse, 1796, Benjamin West (1738-1820, USA). Oil on canvas. 1A

Desk Album: Flower and Bird Paintings (mallow flowers), 18th century, Zhang Ruoai (1713-1746, China). Album leaf, ink and color on paper. 2S

Destruction of the Beast and the False Prophet, 1804, Benjamin West (1738-1820, USA). Oil on panel. 1A 2S

Device, ca. 1961-1962, Jasper Johns (1930, USA). Oil on canvas with wood and metal attachments. 2S

Diary: December 12, 1941, 1980, Roger Shimomura (1939, USA). Acrylic on canvas. 1A* 1B

Die Junge Morgenländerin (The Young Eastern Woman), 1838, Friedrich Amerlin (1803-1887, Austria). Oil on fabric. 1A

Die Sünde kommt in die Welt, 1771-1799, Johann Heinrich Füssli (1741-1825, Switzerland, England). Oil on canvas. 1A 1B

Dreams No. 2, 1965, Jacob Lawrence (1917-2000, USA). Tempera on fiberboard. 2S

Egg Salad, 1850, unknown (unknown, USA). Oil on canvas. 1A 2S

Evocation of Butterflies, c. 1910-1912, Odilon Redon (1840-1916, France). Oil on canvas. 1A* 2R

Evocation of Roussel, ca. 1912, Odilon Redon (1840-1916, France). Oil on canvas. 2S

Fellah Women Drawing Water (Medinet-el-Fayoum), c. 1868-1870, Jean-Leon Gerome (1824-1904, France). Oil on wood. 1A* 2S

Fishermen at Sea, ca. 1913, Henry Ossawa Tanner (1859-1937, USA). Oil on canvas. 2S

Flecks on Foam, ca. 1911-1912, Henry Golden Dearth (1864-1918, USA). Oil on wood. 2S

Flower in Glass, 1606, Ambroisus Bosschaert (1573-1621, Belgium). Oil on copper. 2S

Flowers in a Glass, 1606, Ambroisus Bosschaert (1573-1621, Dutch). Oil on copper. 1A

Francis Bacon as a Child, 1561-1562, Unknown Artist (?, England (?)). Oil on canvas. 1A

From One Night to Another, 1947, Yves Tanguy (1900-1955, France). Oil on canvas. 1A*

Frosty Day, 1915, Alexej von Jawlensky (1864-1941, Russian-German). Oil on canvas board. 2S

Fruit of the Seasons, c. 1860, Frederick Kost (1861-1923, USA). Oil on bed ticking. 1A

Fruits of the Seasons (current place unknown), ca. 1860, Robert Kost (1936-2003, Canada). Oil on canvas. 2S

Girl in a Green Blouse, 1917, Amedeo Modigliani (1884-1920, Italy). Oil on canvas. 2S

Girl in Glitz, 1987, Mary Pratt (1935, Canada). Oil on masonite. 1A

Girls Carrying a Canoe, Vaiala in Samoa, 1891, John La Farge (1835-1910, USA). Watercolor, gouache, and graphite on off-white wove paper. 2S

Glass and Checkerboard, ca. 1917, Juan Gris (1887-1927, Spain). Oil on wood. 2S

Greek Slave, 1870, Jean Leon Gerome (1824-1904, France). Oil on canvas. 1A 1B

Green Landscape, 1971, Gregorie Gillespie (1936-2000, USA). Oil and acrylic on paper collage mounted on wood. 1A 1B* 2S

Haystacks in Brittany, 1890, Paul Gauguin (1848-1903, France). Oil on canvas. 2S

Head of a Damned Soul from Dante's "Inferno", 1770-1778, Johann Heinrich Füssli (1741-1825, Germany). Oil on canvas. 1A 1B

Head of a Damned Soul from Dante's "Inferno", (verso), 1770-1778, Johann Heinrich Füssli (1741-1825, Germany). Oil on canvas. 1A

Henry Pelham (Boy with a Squirrel), 1765, John Singleton Copley (1738-1815, USA). Oil on canvas. 1A

Hercules as Heroic Virtue Overcoming Discord, 1632-1633, Peter Paul Rubens (1577-1640, Flemish). Oil on panel. 1A

Hidden Fortress, 1961, Al Held (1928-2005, USA). Acrylic on canvas. 1A 2S

Hindola Raga, c. late 18th Century, unknown, Pahari, Kangra School, India (unknown, India). Watercolor on paper. 1A* 1B

Homage to Nina Simone, 1965, Bob Thompson (1937-1966, USA). Oil on canvas. 1A* 1B 2S

Interlude, 1960, Milton Avery (1885-1965, USA). Oil on canvas. 2R

Italian Landscape with Umbrella Pines, 1807, Hendrik Voogd (1768-1839, Dutch). Oil on canvas. 2S

Jim, 1930, William H. Johnson (1901-1970, USA). Oil on canvas. 1A 1B

Jove Casts His Thunderbolts at the Rebellious Giants, 1690-1695, Johann Michael Rottmayr (1654-1730, Austria ?). Oil on canvas. 1A

Junior Wells, 1989, Frederick Brown (1945-2012, USA). Oil on linen. 1A* 2S

Kama, Indra, Parvati, and Angels, c. early 17th Century, unknown, Rajasthan, India (unknown, India). Watercolor and gold on paper. 1A

Kedara Ragini, c. 1650, unknown, Rajasthan, Malwa School (unknown, India). Ink and color on paper. 1A

Krishna Longing for Radha, c. 1820-1825, unknown, Pahari, Kangra School, India (unknown, India). Gum tempera and gold on paper. 1A 2S

Lady at Her Toilet, 17th Century, Utrecht School (17 c. , Dutch). Oil on canvas. 1A

Lady Seated, 19th c. , Jules Adolphe Goupil (1839-1883, France). Oil on panel. 1A

Landscape with a Sunlit Stream, ca. 1877, Charles-François Daubigny (1817-1878, France). Oil on canvas. 2S

Landscape with Picnickers and Donkeys by a Gate, ca. 1830-1880, Joseph Paul (1804-1887, Britain). Oil on canvas. 2S

Le Condor Embouteillé ou No. 12, 1942, Paul-Émile Borduas (1905-1960, Canada). Gouache on paper. 1A* 2S

Leisure Hours, 1864, John Everett Millais (1829-1896, England). Oil on canvas. 1A

Little Egypt Condo... New York City, 1987, Jean Lacy (1932-, USA). Collage, xerography, gold leaf, museum board. 1A

Little Painting with Yellow (Improvisation), 1914, Wassily Kandinsky (1866-1944, Russia). Oil on canvas. 2S

Louise de Keroualle, 1671, Peter Lely (Peter van der Faes Lilley) (1618-1680, England). Oil on canvas. 1A

Lucretia, 1534, School of Jan Gossaert (c. 1478-c. 1532, Italy). Oil on panel to canvas transfer. 1A 1B

Madame Cézanne (Hortense Fiquet, 1850-1922) in a Red Dress, 1888-90, Paul Cézanne (1839-1906, France). Oil on canvas. 2S

Magnolia Grandiflora, ca. 1885-1895, Martin Johnson Heade (1819-1904, USA). Oil on canvas. 2R

Mandala of Shiva and Shakti, c. 1700-1799, unknonwn, Shah Dynasty, Bhaktapur, Nepal (unknown, Nepal). Ground mineral pigment on cotton. 1A

Maple, Chrysanthemum, and Bush-clover, mid 17th Century, Kitagawa Sosetu (mid 17th Century, Japan). Ink, color, and gold on paper; mounted as a four-panel wooden screen. 1A 1B 2R

Mars and Venus, Allegory of Peace, 1770, Louis-Jean-Francois Lagrenee (1725-1805, France). Oil on canvas. 1A

Medusa, 1892, Alice Pice Barney (1857-1931, USA). Pastel on canvas. 1A* 1B 2S

Midsummer's Dream #173 from the series Cosmic Dance No. 3, 1979, Sheila Eaton Isham (1927, USA). Acrylic and oil on linen. 2S

Mount Vesuvius at Midnight, 1868, Albert Bierstadt (1830-1902, USA). Oil on canvas. 1A* 2S

Mountain Farm in Snow, c. 1913, Preston Dickenson (1891-1930, USA). Oil on canvas. 1A 1B

Mrs. Benjamin Hallowell, c. 1766, John Singleton Copley (1738-1815, USA). Oil on canvas. 1A

Murnau, 1910, Alexej von Jawlensky (1864-1941, Russian-German). Oil on handboard. 2S

Niagara Falls from Table Rock, 1835, Samuel Finley Breese Morse (1791-1872, USA). Oil on canvas. 1A 1B 2S

On The Beach, 1945, Alfred Pellan, 1906-1988 (1906-1988, Canada). Oil on canvas. 1A

Onions, 1881, Pierre-Auguste Renoir (1841-1919, France). Oil on canvas. 2S

Oranges in Tissue Paper, c. 1890, William J. Mc. Closkey (1859-1941, USA). Oil on canvas. 1A

Pandora, 1941, Ben Benn (1884-1983, USA). Oil on canvas. 2S

Panel for a Screen: Children Frightened by a Rabbit, ca. 1876, Albert Pinkham Ryder (1847-1917, USA). Oil on gilded leather mounted on canvas. 2S

Panel for a Screen: Children Playing with a Rabbit, ca. 1876, Albert Pinkham Ryder (1847-1917, USA). Oil on gilded leather mounted on canvas. 2S

Passage Violet, 1967, Fernand Leduc (1916-2014, Canada). Acrylic on canvas. 1A 1B

Pastoral Landscape: The Roman Campagna, ca. 1639, Claude Lorrain (Claude Gellée) (1604/5?-1682, Italy). Oil on canvas. 2S

Peasant Girl with Sheep, 19th-20th Century, Julien Dupre (1851-1910, France). Oil on canvas. 1A 2S

Political Drama, 1914, Robert Delaunay (1885-1941, France). Oil and collage on cardboard. 2S

Portrait of a Child, 1805, Jean Baptiste Greuze (1725-1805, France). Oil on canvas. 1A 1B*

Portrait of a Farmer's Wife, 1954, Robert Gwathmey (1903-1988, USA). Oil on canvas. 1A

Portrait of a Lady, 1771, John Singleton Copley (1738-1815, USA). Oil on canvas. 1A

Portrait of a Woman, 1660, Jan Mytens (1614-1670, Dutch). Oil on canvas. 1A

Portrait of a Woman, 1851, Jean-Leon Gerome (1824-1904, France). Oil on canvas. 1A

Portrait of a Woman in a Rose-Colored Gown (La Dame en Rose), 18th Century, Maurice Quentin de la Tour (1704-1788, France). Pastel on paper. 1A

Portrait of Clémentine (Mrs. Alphonse) Karr, 1845, Henri Lehmann (1814-1882, France). Oil on canvas. 1A

Portrait of Harriet Campbell, 1815, Ammi Phillips (1788-1865, USA). Oil on canvas. 1A

Portrait of Madame X, 1907, Giovanni Boldini (1845-1931, Italy). Oil on panel. 1A

Portrait of Mlle. Lange as Danae, 1799, Anne-Louis Girodet de Roucy Trioson (1767-1824, France). Oil on canvas. 1A

Portrait of Walter Pach, 1932-1949, Jacques Villon (1875-1963, France). Oil on canvas. 1A 1B 2S

Prometheus Bound, 1847, Thomas Cole (1801-1848, USA). Oil on canvas. 1A 2S

Red Sunset, Old Pond Concerto, 1972, Alma Thomas (1891-1978, USA). Acrylic on canvas. 1A 2R

River Valley, ca. 1626-1630, Hercules Segers (1589-1638, Dutch). Oil on panel. 2S

Rose's Last Summer, 1985, Gael Stack (1942-, USA). Oil on canvas. 1A* 1B

Russian Country Fair, 1944, Raisa Robbins (20th Century, USA). Oil on canvas. 1A 1B

Rustam's Seventh Course: He Kills the White Div: Illustration from the Manuscript, the "Tahmasp Shahnama" of Firdausi, 1520s-1540s, Mir Musavvir (1533-1609, Persia). Watercolor, gold and silver on paper. 1A

sans titre no3, 1948, Charles Daudelin (1920-2001, Canada). Gouache on paper. 1A 1B

Saturday Nite, 1971, Clementine Hunter (1884-1988, USA). Oil on canvas board. 1A 2S

Scene during the Eruption of Vesuvius, c. 1827, Joseph Franque (1774-1833, France). Oil on canvas. 1A

Seaside (July: Specimen of a Portrait), 1887, James Tissot (1836-1902, France). Oil on fabric. 1A

Seated African Woman, c. 1860, Henri Regnault (1843-1871, France). Oil on canvas. 1A* 1B

Self-Portrait as a Yawning Man, c. 1783, Joseph Ducreux (1735-1802, France). Oil on canvas. 1A

Self-Portrait with Yellow Table, 1981, Louisa Matthiasdottir (1917-2000, USA). Oil on canvas. 1A 1B 2R

Shore and Surf, Nassau, 1899, Winslow Homer (1836-1910, USA). Watercolor and graphite on off-white wove paper. 2S

Simplon Pass, 1911, John Singer Sargent (1856-1925, USA). Oil on canvas. 2S

Sioux Worshiping at the Red Boulders, ca. 1837-1839, George Catlin (1796-1872, USA). Oil on canvas. 2S

Sita in the Garden of Lanka, From the Ramayana epic of Valmiki, c. 1725-1727, Ramayana Pahari Paintings; western Himalayan kingdom of Guler (unknown, India). Color and gold on paper. 1A 2R

Sleigh Ride, ca. 1890-1895, Winslow Homer (1836-1910, USA). Oil on canvas. 2R

Somewhere in America, 1934, Robert Brackman (1898-1980, USA). Oil on canvas. 1A

Still Life, 1921, Amédée Ozenfant (1883-1966, France). Oil on canvas. 1A 1B

Still Life with Flowers and Fruit, 1869, Claude Monet (1840-1926, France). Oil on canvas. 1A 2S

Still Life with Flowers, Shells, A Shark's Head, and Petrifications, 1819, Antoine Berjon (1754-1843, France). Oil on canvas. 1A* 2R

Still Life with Oysters and Grapes, 1653, Jan Davidsz Heem (1606-1684, Dutch). Oil on panel. 1A 2S

Study for "A Sunday on La Grande Jatte", 1884, Georges Seurat (1859-1891, France). Oil on canvas. 2S

Study for Delfina, 1967, Jack Youngerman (1926-2020, USA). Lithograph on paper. 2S

Study for Eagle Head, Manchester, Massachusetts, 1869, Winslow Homer (1836-1910, USA). Oil on wood. 2S

Summer, 1890, Thomas Wiolmer Dewing (1851-1938, USA). Oil on canvas. 1A 1B 2R

Sunrise on the Matterhorn, after 1875, Albert Bierstadt (1830-1902, German American). Oil on canvas. 2S

Tahkt-I-Sulayman Variation II, 1969, Frank Stella (1936, USA). Acrylic on canvas. 2S

The Abbatial House, Abbey of St. Ouen, Rouen, 1824-32, John Sell Cotman (1782-1842, Britain). Graphite and watercolor, heightened with bodycolor and with scratching out. 2S

The Angels Appearing to Abraham, 1750s, Fr. sco Guardi (1712-1793, Italy). Oil on canvas. 1A

The Apple Bloom, 1903, Robert Vonnoh (1858-1933, USA). Oil on canvas. 2S

The Battle of the USS "Kearsarge" and the CSS "Alabama", 1864, Édouard Manet (1832-1883, France). Oil on canvas. 2S

The Beeches, 1845, Asher B. Durand (1796-1886, USA). Oil on canvas. 1A 1B

The Betrayal of Christ, c. 1618-1620, Anthony Van Dyck (1599-1641, Belgium). Oil on canvas. 1A

The Blue Dress (or A Duchess), 1861, Alfred Stevens (1823-1906, Belgium). Oil on panel. 1A

The Bouquet, c. 1870, Raimundo Madrazo y Garreta (1841-1920, Spain). Oil on panel. 1A

The Chariot of Apollo, 1905-16, Odilon Redon (1840-1916, France). Oil on canvas. 2S

The Chase, 1999, Susan Rothenberg (1945-2020, USA). Oil on canvas. 2S

The Church of Souain, 1917, Félix Vallotton (1865-1925, Switzerland). Oil on canvas. 2S

The Comtesse d'Haussonville (Louise de Broglie), 1845, Jean-Auguste-Dominique Ingres (1780-1867, France). Oil on canvas. 1A

The Dead Soldier, 1789, Joseph Wright (1734-1797, Great Britain). Oil on canvas. 1A 2S

The Death of Lucretia, c. 1735-1737, Ludovico Mazzanti (1686-1775, Italy). Oil on canvas. 1A

The Death of Sardanapalus, 1844, Ferdinand-Victor-Eugene Delacroix (1798-1863, France). Oil on canvas. 1A 1B

The Flagellation, c. 1912-1925, Georges Rouault (1871-1958, France). Oil on canvas. 1A 1B

The Four Seasons (Part 1: Spring), c. 1755, Francois Boucher (1703-1770, France). Oil on canvas. 1A

The Good Shepherd, ca. 1918, Henry Ossawa Tanner (1859-1937, USA). Oil on canvas on particle board. 2S

The Great Goddess Durga Slaying the Buffalo Demon (Mahishasuramardini), c. 1750, unknown (unknown, unknown). Opaque watercolor with gold-and silver-colored metallic paint on paper. 1A 1B

The Harbor, ca. 1943, Josef Presser (1907-1967, Poland, USA). Oil on wood. 2S

The Madness of the Gopis, c. 1750, unknown, Rajasthan, India (unknown, India). Watercolor with gold on paper. 1A

The Magic Room, 1994, Patssi Valdez (1951-, USA). Acrylic on canvas. 1A* 2R

The Martyrdom of St. Cecilia, c. 1610, Carlo Saraceni (1579-1620, Italy). Oil on canvas. 1A

The Morning Visit or Confidences, c. 1870, Raimundo Madrazo y Garreta (1841-1920, Spain). Oil on canvas. 1A 1B

The Nightmare, 1781, Henry Fuseli (1741-1825, Swiss). Oil on canvas. 1A*

The Orange Market, Venice, 1876, James Holland (1799-1870, Britain). Watercolor and gouache over graphite on paper. 2S

The Protagonist of an endless Story, 1993, Angel Rodríguez-Díaz (1955, Puerto Rico/USA). Oil on canvas. 1A* 1B

The Questioner of the Sphinx, 1863, Elihu Vedder (1836-1923, USA). Oil on canvas. 1A

The Rape of Europa, c. 1726-1727, Noël-Nicolas Coypel (1690-1734, France). Oil on canvas. 1A 1B 2S

The Reaper with a Sickle, 1838, Jean-Baptiste Camille Corot (1796-1875, France). Oil on canvas. 1A 1B

The Red Path, St. Prex, 1915, Alexej von Jawlensky (1864-1941, Russian-German). Oil on paper on cardboard. 2S

The Roman Daughter, 1811, Rembrandt Peale (1778-1860, USA). Oil on canvas. 1A 1B

The Salutation of Beatrice, 1859, Dante Gabriel Rossetti (1828-1882, Britain). Oil on wood. 1A

The Scout: Friends or Foes, ca. 1900-1905, Frederic Remington (1861-1909, USA). Oil on canvas. 1A

The Street Pavers, 1914, Umberto Boccioni (1882-1916, Italy). Oil on canvas. 2S

The Veteran in a New Field, 1865, Winslow Homer (1836-1910, USA). Oil on canvas. 2S

The Visit-Couple and Newcomer, 1922, Ernst Ludwig Kirchner (1880-1938, Germany). Oil on canvas. 2S

The Wave, c. 1869-1870, Gustave Courbet (1819-1877, France). Oil on canvas. 1A 2S

The Wheel of the Seasons, 1957, Charmion Von Wiegand (1896-1983, USA). Oil on canvas. 2S

The Wreck, 1854, Eugene-Louis-Gabriel Isabey (1804-1886, France). Oil on canvas. 1A

Thirty-Six Immortal Poets, c. 1740-1750, Kagei Tatebayashi (c. 1504-c. 1589, Japan). Two-fold screen, ink and color on paper. 1A

Thorn Trees, 1945, Graham Vivian Sutherland (1903-1980, Britain). Oil on cardboard. 2R

Three Dancers, c. 1940, William H. Johnson (1901-1970, USA). Oil on burlap. 1A 1B

Tomatoes, Fruit, and Flowers, c. 1860, unknown, American (unknown, USA). Oil on canvas. 1A 1B 2S

Tommaso Portinari, 1472, Hans Memling (1465-1494, Netherlands). Oil on wood. 1A

Triumphant Child, 1946, Walter Quirt (1902-1968, USA). Oil on canvas. 1A 2S

Trompe-l'Oeil Still Life with a Flower Garland and a Curtain, 1658, Adriaen van der Spelt (1630-1673, Dutch). Oil on panel. 1A

Turning Point of Thirst, 1934, Victor Brauner (1903-1966, Romania). Oil on canvas. 1A 1B

Two Nudes [obverse], 1907, Ernst Ludwig Kirchner (1880-1938, Germany). Oil on canvas. 2S

Unidentified Raga, c. 1775, unknown, Indian (unknown, India). Watercolor and gold on paper. 1A

Untitled XIII, 1985, Willem de Kooning (1904-1997, Dutch). Oil on canvas. 2S

Venus, c. 1518, Lucas (the Elder) Cranach (1472-1553, Germany). Oil on lime. 1A

View of Houses in Delft, Known as 'The Little Street', ca. 1658, Johannes Vermeer (1632-1675, Dutch). Oil on canvas. 2S

View of Rotterdam, ca. 1867, Johan Barthold Jongkind (1819-1891, Dutch). Watercolor. 2S

View on Catskill Creek, 1867, John William Hill (1812-1879, Britain). Watercolor, gouache, and graphite on off-white wove paper. 2S

Vision of the Sage Markandeya, c. 1775-1800, unknown, Himachel Pradesh reg., India (unknown, India). Watercolor and gold on paper. 1A

Watson and the Shark, 1782, John Singleton Copley (1738-1815, USA). Oil on canvas. 1A

Way Down Blue, 1945, Balcomb Greene (1904-1990, USA). Oil on canvas. 2S

West Point, Prout's Neck, 1900, Winslow Homer (1836-1910, USA). Oil on canvas. 2S

Woman and Flowers (Opus LIX), 1868, Sir Lawrence Alma-Tadema (1836-1912, Dutch). Oil on panel. 1A 1B

Women Sewing, ca. 1912, Édouard Vuillard (1868-1940, France). Glue on paper on canvas. 2S

Yama, King of Hell, c. 1800, unknown (unknown, Tibet). Watercolor on cotton. 1A

Young Girl Counting, Late 19th Century, Eugène Carrière (1849-1906, France). Oil on canvas. 1A

Young Woman with a Fan, c. 1754-1756, Pietro Rotari (1707-1762, Italy). Oil on canvas. 1A

Young Woman with a Turban, c. 1780, Jacques Louis David (1748-1825, France). Oil on canvas. 1A

Young Women Jumping Rope, c. 1942-1944, Rufino Tamayo (1899-1991, Mexico). Oil on canvas. 1A

Tama River in Musashi Province (Bushū Tamagawa), from the series Thirty-six Views of Mount Fuji (Fugaku sanjūrokkei), ca. 1830-32, Katsushika Hokusai (1760-1849, Japan). Polychrome woodblock print; ink and color on paper. 2S

Cultural and Lifestyle Questionnaire, Original German

Fragebogen zu Kultur und Lebensstil

In diesem Fragebogen bitten wir Sie, 20 Fragen über Ihren Geschmack, kulturellen Hintergrund und Lebensstil zu beantworten. Die Antworten sollen so ehrlich wie möglich sein und so viele Details wie möglich enthalten. Es gibt keine richtigen oder falschen Antworten. Falls eine Frage nicht auf Sie zutrifft, können Sie sie überspringen. Die Angaben, die Sie in diesem Fragebogen machen, sind vertraulich und werden an niemanden weitergegeben.

Erinnerungen:

1. Bitte nennen Sie die Stadt, in der Sie aufgewachsen sind und beschreiben Sie einen Ort dort, an den Sie sich lebhaft erinnern.

2. Was war Ihr bisher bemerkenswertester Urlaub? (Bitte nennen Sie den Namen der Stadt/Städte, einige Orte, die Sie dort besucht haben, und erklären Sie kurz, wieso dieser Urlaub solch einen Eindruck hinterlassen hat.)

Interessen und Lebensstil:

Orten

3. In welchen anderen Städten (und jeweiligen Ländern) haben Sie außerdem Urlaub gemacht? (max. 5)

4. Gibt es Orte, die Sie von Fotos oder Videos kennen und die Sie gerne besuchen würden? Welche Orte sind das?

5. Welche Orte in der Nähe Ihres Wohnortes sind für Sie von Bedeutung? (Beispielsweise: Sehenswürdigkeiten, Parks, Nachbarschaften, Restaurants, usw.) An welchen Ort denken Sie zuerst, wenn Sie einen Spaziergang machen wollen?

6. Wenn Sie einen Ort in Ihrem Heimatland empfehlen sollten, den man unbedingt besuchen sollte, welcher wäre das?

7. Welche Art von Umgebung bevorzugen Sie, wenn Sie in Kontakt mit der Natur kommen wollen? Sie können mehr als eine Antwort geben. (zum Beispiel: Strand, Wald, Berge, See, Wüste usw.)

Interessen

8. Mit welchen sozialen, kulturellen oder religiösen Gruppen identifizieren Sie sich? Sie können mehrere Antworten geben. (Einige Beispiele: ”Ich stehe wirklich auf Swing-Tanz”, ”Ich bin ein LGBTQ-Aktivist”, ”Ich bin ein Hacker”, ”Ich bin ein frommer Katholik”)

9. Welche Aktivitäten kommen Ihnen in den Sinn, wenn Sie an einen verregneten Tag denken?

10. Welche anderen Kulturen sprechen Sie am meisten an (z. B. ihre Gewohnheiten, Kunst, Essen usw.)?

11. Was sind Ihre Hobbys (z. B. Münzen oder Spielzeug sammeln, Basteln, Kochen, Lesen usw.)?

12. Üben sie Sport oder andere körperliche Aktivitäten aus? Welche? Gibt es außerdem Sportarten, die Sie verfolgen?

13. Mögen Sie Filme oder Serien? Nennen Sie einige, die Sie für bemerkenswert halten.

14. Haben Sie Freude an der Kunst (einschließlich Malerei, Musik, Tanz, Theater usw.)? Können Sie eine*n Interpret*in nennen, den*die Sie für herausragend halten?

15. Spielen Sie ein oder mehrere Instrument/e?

16. Mögen Sie Museen? Gibt es welche, die Sie besonders bemerkenswert finden/die Sie besonders empfehlen würden?

17. Gibt es Bücher, die einen besonderen Eindruck bei Ihnen hinterlassen haben? Was hat Ihnen daran besonders gefallen?

18. Nennen Sie mindestens 3 Gerichte, mit denen Sie Erinnerungen verbinden. Welche Küchen mögen Sie besonders?

19. Haben/Hatten Sie Haustiere? Wenn ja, welche? Was sind Ihre Lieblingstiere?

20. Wie würden Sie Ihren Kleidungsstil beschreiben? Gibt es Marken, deren Kleidungsstücke Sie häufig kaufen?

Cultural and Lifestyle Questionnaire, English translation

In this form, we ask you to answer 20 questions about your tastes, cultural background and lifestyle. The answers should be as honest as possible, and contain as much detail as possible. This will help us understand your preferences and personality. There are no right or wrong answers and in case the question doesn’t apply to you, you can skip it. The information provided in this form is confidential and will not be shared with anyone.

Memories:

1. Can you name the city where you grew up and describe a place there that you remember vividly?

2. What was your most remarkable holiday until now? (Please, include the name of city/cities, some places you visited there, and why it left an impression).

Main interests and lifestyle:

Places

3. Can you mention other cities, and their respective countries, that you have been for holiday/vacation? (max. 5)

4. Are there places that you know from photos or videos that you would like to visit? Which ones?

5. What places near where you live are significant to you? (For example: landmarks, parks, neighborhoods, restaurants, etc.) Which is the first place you think about, when you want to go for a walk?

6. If you could recommend a place to visit in your homeland, what would it be?

7. When you want to be in contact with nature, what type of settings do you prefer? More than one answer can be provided (For example: beach, forest, mountain, lake, desert, etc.).

Interests

8. What social, cultural, or religious groups do you identify with? (Some examples: “I’m really into swing dancing”, “I’m an LGBTQ activist”, “I am a hacker”, “I am a devout Catholic”). There can be multiple answers.

9. What activities come to your mind when you think about a rainy day?

10. Which other cultures appeal to you the most (e. g. their habits, arts, food, etc.)?

11. What are your hobbies (e. g. collecting coins or toys, crafts, cooking, reading, etc.)?

12. Do you practice any sports or any physical activities? Which ones? Additionally, are there any that you follow?

13. Do you enjoy movies or series? Name some that you consider noteworthy.

14. Do you enjoy the arts (including paintings, music, dance, theater, etc.)? Can you name an artist or performer that you consider outstanding?

15. Do you play any instruments?

16. Do you enjoy museums? Are there any the stand out to you?

17. Are there books that left an impression on you? What is the main thing you liked about them?

18. List at least 3 dishes that are associated with a memory. And what are your favorite cuisines?

19. Do you have/had pets? What type? What are your favorite animals?

20. How would you define your dressing style? Are there any brands that you usually buy more frequently than others?

Full Instructions

Instructions for aesthetic appeal, Experiments 1A and 2:

Stellen Sie sich vor, die Bilder, die sie sehen seien Kunstwerke, die ein Kunstmuseum möglicherweise erwerben möchte. Der Kurator will wissen, welche Kunstwerke am bewegendsten sind, basierend darauf, wie stark Sie als Person auf die Werke reagieren. Ihre Aufgabe ist es, aus dem Bauch heraus zu antworten, je nachdem wie schön, fesselnd oder ausdrucksstark Sie die Werke finden. Beachten Sie: Die Kunstwerke können die gesamte Bandbreite von ”schön” bis ”seltsam” oder gar ”hässlich” abdecken und können Ihnen mehr oder weniger bekannt vorkommen. Wichtig ist, dass Sie angeben, welche Kunstwerke Sie ausdrucksstark, ansprechend oder tiefsinnig finden.

Imagine that the images you see are artworks that may be acquired by a museum of fine art. The curator wants to know which artworks are the most aesthetically pleasing based on how strongly you as an individual respond to them. Your job is to give your gut-level response based on how much you find the artworks beautiful, compelling, or powerful. Note: The artworks may cover the entire range from "beautiful" to "strange" or even "ugly", and may be more or less familiar. We ask that you respond on the basis of how much this artwork "moves" you. What is most important is for you to indicate what works you find powerful, pleasing, or profound.

Instructions for self-relevance (all experiments):

Selbst-Relevanz ist das Ausmaš, in dem sich etwas auf Sie, Ihre Erfahrungen oder Ihre Identität bezieht. Dies sind die Dinge und Ereignisse, die Sie als Person definieren. Wenn Sie beispielsweise einen neuen Freund kennenlernen, sind dies die Sachen, die sie über sich erzählen würden, um ihm ein Gefühl dafür zu geben, wer Sie sind. Ihre Aufgabe ist es, jedes Bild anzuschauen und aus dem Bauch heraus zu entscheiden, wie sehr das abgebildete Kunstwerk sich auf Sie, Ihre Interessen und Hobbies, Ihre Persönlichkeit, auf Orte und Menschen, die Sie kennen, oder auf Ereignisse in Ihrem eigenen Leben bezieht. Beachten Sie: Die Kunstwerke können die gesamte Bandbreite von ”schön” bis ”seltsam” oder gar ”hässlich” abdecken und können Ihnen mehr oder weniger bekannt vorkommen. Wichtig ist, dass Sie angeben, inwieweit der Inhalt des Kunstwerkes für Sie persönlich relevant ist. So kann es sein, dass ein hässliches Bild für Sie persönlich relevant ist, oder ein ansprechendes Kunstwerk für Sie persönlich nicht relevant ist. Ebenso kann sich ein Kunstwerk, das Ihnen bekannt vorkommt, für sie selbst-relevant anfühlen oder eben auch nicht.

Self-relevance is the extent to which something relates to you, your experiences or your identity. These are the things and events that define you as a person. For example, when you are getting to know a new friend, these are things that you might tell them about to give them a sense for who you are. Your job in this task is to look at each image and give your gut-level response based on how much you think the depicted artwork relates to you, your interests and hobbies, your personality, to places or people you know, or to events in your personal life. Note: The artworks may cover the entire range from "beautiful" to "strange" to even "ugly", and may be more or less familiar. We ask that you respond on the basis of how much the content of the artwork is relevant to you personally. Thus, you may find an ugly artwork personally relevant, or a pleasing artwork to not be relevant to you. Similarly, a familiar painting may or may not feel self-relevant to you.

Instructions for familiarity:

HINWEIS/BITTE BEACHTEN SIE: Alle Bilder wurden Ihnen in den vorherigen Blöcken dieses Experiments gezeigt. Dies ist jedoch nicht das, wonach wir fragen. Wir bitten Sie zu bewerten, ob die Bilder vor diesem Experiment erkennbar oder Ihnen vertraut sind.

Bitte wählen Sie ”Erkenne eindeutig”, wenn Sie sicher wissen, dass Sie das Kunstwerk (oder ein Bild davon) schon einmal gesehen haben —wenn Sie sich sehr sicher sind und sich vielleicht sogar daran erinnern, es schonmal gesehen

zu haben. Bitte wählen Se ”Vertraut”, wenn sie das Gefühl haben, dass Sie das Kunstwerk (oder ein Bild davon) schon einmal gesehen haben oder wenn Ihnen der Inhalt des Kunstwerkes vertraut erscheint. Wählen Sie diese Antwort, wenn sich das Kunstwerk vertraut anfühlt, Sie sich aber unsicher sind, ob Sie es schon einmal gesehen haben —oder wo und wann das gewesen sein könnte. Bitte wählen Sie ”Unbekannt”, wenn Ihnen der Inhalt des Bildes unbekannt ist und Sie sich sehr sicher sind, dass Sie das Kunstwerk (oder ein Bild davon) noch nie zuvor gesehen haben.

PLEASE NOTE: All the images were shown to you in the previous blocks of this experiment. However, this is not what we are asking about. We ask you to rate whether the images are recognizable or familiar to you prior to this experiment.

Please select ”Recognize clearly” if you know for sure that you have seen the work of art (or a picture of it) before —if you are very sure and perhaps even have a specific memory of seeing it. Please select ”Familiar” if you have the feeling that you have seen the work of art (or a picture of it) before or if the content of the work of art appears familiar to you. Choose this answer if the artwork feels familiar, but you are unsure whether you've seen it before —or where and when it might have been. Please select ”Unknown” if you do not know the content of the picture and are very sure that you have never seen the work of art (or a picture of it) before.

Instructions for ”Beauty” (Experiment 1B):

In this question you are to rate how beautiful you found the image. Note that we are not asking how much it moves you, but whether you feel like it is attractive, and you get the feeling of beauty.

Instructions for ”Being Moved” (Experiment 1B):

In this question you are to rate how moved you were by the image. Note that we are not asking how conventionally beautiful, ugly or interesting the image is, what matters is whether you feel personally touched.

References

Azen, R. and Budescu, D. V. (2003). The dominance analysis approach for comparing predictors in multiple regression. *Psychological Methods*, 8:129–148.

Geller, H. A., Bartho, R., Thömmes, K., and Redies, C. (2022). Statistical image properties predict aesthetic ratings in abstract paintings created by neural style transfer. *Frontiers in Neuroscience*, 16.

Kotabe, H. P., Kardan, O., and Berman, M. G. (2017). The nature-disorder paradox: A perceptual study on how nature is disorderly yet aesthetically preferred. *Journal of Experimental Psychology: General*, 146:1126–1142.

Lee, J., Cho, S., and Beack, S.-K. (2018). Context-adaptive entropy model for end-to-end optimized image compression. *arXiv preprint arXiv:1809.10452*.

Luchman, J. (2022). domir: Tools to support relative importance analysis.

Martinez, J. E., Funk, F., and Todorov, A. (2020). Quantifying idiosyncratic and shared contributions to judgment. *Behavior Research Methods*, 52:1428–1444.

Mathôt, S., Schreij, D., and Theeuwes, J. (2012). Opensesame: An open-source, graphical experiment builder for the social sciences. *Behavior Research Methods*, 44:314–324.

Needell, C. D. and Bainbridge, W. A. (2022). Embracing new techniques in deep learning for estimating image memorability. *Computational Brain and Behavior*, 5:168–184.

Pan, J., Ferrer, C. C., McGuinness, K., O'Connor, N. E., Torres, J., Sayrol, E., and Giro-i Nieto, X. (2017). Salgan: Visual saliency prediction with generative adversarial networks. *arXiv preprint arXiv:1701.01081*.

Redies, C., Grebenkina, M., Mohseni, M., Kaduhm, A., and Dobel, C. (2020). Global image properties predict ratings of affective pictures. *Frontiers in psychology*, 11:953.

Sutherland, C. A., Burton, N. S., Wilmer, J. B., Blokland, G. A., Germine, L., Palermo, R., Collova, J. R., and Rhodes, G. (2020). Individual differences in trust evaluations are shaped mostly by environments, not genes. *Proceedings of the National Academy of Sciences of the United States of America*, 117:10218–10224.

Uran, C., Peter, A., Lazar, A., Barnes, W., Klon-Lipok, J., Shapcott, K. A., Roese, R., Fries, P., Singer, W., and Vinck, M. (2022). Predictive coding of natural images by v1 firing rates and rhythmic synchronization. *Neuron*, 110(7):1240–1257.

Wagatsuma, N., Hidaka, A., and Tamura, H. (2021). Correspondence between monkey visual cortices and layers of a saliency map model based on a deep convolutional neural network for representations of natural images. *Eneuro*, 8(1).
